# SVhound: detection of regions that harbor yet undetected structural variation

**DOI:** 10.1186/s12859-022-05046-6

**Published:** 2023-01-20

**Authors:** Luis F. Paulin, Muthuswamy Raveendran, R. Alan Harris, Jeffrey Rogers, Arndt von Haeseler, Fritz J. Sedlazeck

**Affiliations:** 1 Center for Integrative Bioinformatics Vienna, Max Perutz Labs, University of Vienna, Medical University of Vienna, Vienna, Austria; 2 Human Genome Sequencing Center, Baylor College of Medicine, Houston, TX USA; 3 Department of Molecular and Human Genetics, Baylor College of Medicine, Houston, TX USA; 4 Faculty of Computer Science, University of Vienna, Vienna, Austria

**Keywords:** Structural variation, SV discovery, Diversity, Population, Sequencing

## Abstract

**Background:**

Recent population studies are ever growing in number of samples to investigate the diversity of a population or species. These studies reveal new polymorphism that lead to important insights into the mechanisms of evolution, but are also important for the interpretation of these variations. Nevertheless, while the full catalog of variations across entire species remains unknown, we can predict which regions harbor additional not yet detected variations and investigate their properties, thereby enhancing the analysis for potentially missed variants.

**Results:**

To achieve this we developed SVhound (https://github.com/lfpaulin/SVhound), which based on a population level SVs dataset can predict regions that harbor unseen SV alleles. We tested SVhound using subsets of the 1000 genomes project data and showed that its correlation (average correlation of 2800 tests r = 0.7136) is high to the full data set. Next, we utilized SVhound to investigate potentially missed or understudied regions across 1KGP and CCDG. Lastly we also apply SVhound on a small and novel SV call set for rhesus macaque (*Macaca mulatta*) and discuss the impact and choice of parameters for SVhound.

**Conclusions:**

SVhound is a unique method to identify potential regions that harbor hidden diversity in model and non model organisms and can also be potentially used to ensure high quality of SV call sets.

**Supplementary Information:**

The online version contains supplementary material available at 10.1186/s12859-022-05046-6.

## Background

The advent of next generation sequencing has enabled us to characterize genomic variations between and within species on an unprecedented scale [1, 2]. This has produced various novel insights based on sequence complexity and previously underestimated genomic variability between individuals within the same species [3]. Since then, reports have described an ever-increasing number of novel genomic variations and their associated allele frequency estimates [3–8]. These findings are important for many fields in research and clinical applications, ultimately providing a better understanding of phenotype to genotype relationships [1, 9, 10].

Over the past years, genomic studies emerged targeting even higher sample numbers to obtain deeper insights into allele frequencies and diversity (genomic variation) among humans or other species [3–5, 11]. One of the spearheading projects in the past years was the 1000 Genomes Project (1KGP), which cataloged single nucleotide variations (SNV) and structural variations (SV) among 2504 individuals from different ethnicities around the world [3]. While it is clear that the 1KGP catalog is incomplete, it is still one of the most valuable datasets and it is widely used as control data [3]. More recent initiatives such as gnomadSV investigated the presence of SVs across 14,891 human genomes and thus deepened our knowledge of human genome diversity (discovering ~ 445 k SVs) and allele frequencies that are important for multiple aspects [5], such as ranking and annotating variations or identifying population structure. However, even larger studies are underway (e.g. Topmed [12], CCDG [11]) that will identify many new SNVs/SVs in presumably healthy individuals and lead to even more robust ancestry specific allele frequencies and also to a better understanding of variability with respect to diseases [13].

The detection of genomic variations is often promoted by technological and methodological advances in computational methods [9, 14]. As an example, microarrays enabled the first identification of so-called large copy number variations (CNV), in the range of kbp to Mbp, at scale [15]. Subsequently, short read sequencing technologies (whole exome or whole genome sequencing) detected these large alterations and SNVs simultaneously. Many developments in computational methods led to a better characterization of large events (e.g. CNV of multiple kbp) and identification of even more complex structural variations [9]. The continuous advance of better benchmark datasets (e.g. GIAB [16]) and software will lead to many newly identified variations in currently hard to assess regions (e.g. dark regions) of the genome [17].

Despite these developments and the increased number of studies sequencing hundreds to thousands of humans, we still expect an unknown number of undetected genomic variations including rare or even common alleles. This is especially true for ethnicities that have not yet been extensively sequenced (e.g. non-European) [7]. Thus, the questions arise: which genomic regions carry novel yet undetected variations in our enlarged datasets? Can we predict such genomic regions based on existing sequencing data, and if so where are these regions located in the genome and what else can we learn about the mechanisms generating SVs?

To address these questions, we utilized large genomic SV datasets from the 1KGP [3] and CCDG [4] cohorts and applied a population genetic approach that computes the likelihood to observe novel genomic variations, if we had sequenced more individuals. To this end we developed SVhound, which scans the genome for regions of hidden diversity. Thus, by continuing sequencing of a certain population one can expect to find new alleles in this population which we refer to clairvoyant SV (clSV). This is to better distinguish clSV from novel or additional SV that are often reported by e.g. long read sequencing of the same sample set. In the following we demonstrate the predictive power of SVhound based on the analysis of the 1KGP dataset. Next, we applied SVhound to the CCDG cohort composed of a collection of 19,652 human samples [4]. Finally, SVhound is applied to uncover regions of undetected genomic variability in genomes from 150 rhesus macaques (Macaca mulatta), an important model species for human diseases and evolutionary studies. Currently, little is known about SVs in rhesus macaques [18, 19]. SVhound introduces a novel prediction framework to identify genomic regions that are lacking genotypes from current large-scale sequencing and studies the properties of these regions and their potential role. Finally, we provide an easy to use R package freely available at https://github.com/lfpaulin/SVhound.

## Results

### Statistical identification of highly variable genomic regions in the human population

Here we present SVhound, a tool to predict potential regions where additional Structural Variation (SV, defined as genomic variation greater than 50 bp) can be expected if more genomes were sequenced.

In short, SVhound partitions a genome into non-overlapping windows. For each window, SVhound counts the number of different SV-alleles that occur in a sample of *n* genomes (see “Methods”). Based on the number of distinct SV-alleles, SVhound predicts regions that can potentially harbor new structural variants (clairvoyant SV, clSV) by estimating the probability of observing a new SV-allele (see “Methods”). Note these are not SV that are detected within the same sample set by deeper coverage or utilization of long reads, but SV that belong to not yet sequenced samples. Thus, clairvoyant SVs (clSVs) are defined as previously undetected SV of unknown genotype. SVhound assigns probabilities to each region to find a clSV. Thus, regions with a high probability will produce more SV if more samples are sequenced.

**Fig. 1:**
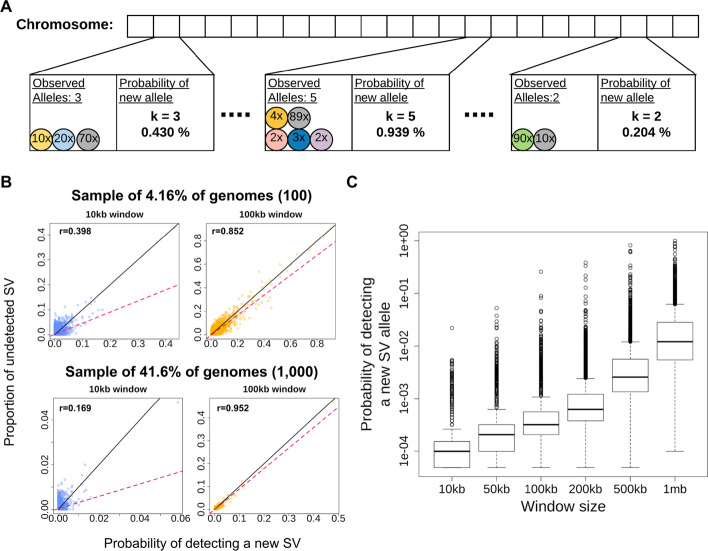
Overview and evaluation of SVhound based on 1000 genomes data set. **A** Computing the probabilities of detecting new SV-alleles in a window. First, the chromosome is divided into non overlapping windows. For each window the number of distinct observed SV-alleles is counted and the diversity parameter is estimated Eq. 2 (see “Methods”). Finally, the probability of detecting a clairvoyant SV (clSV) ($p_{new}$) for each particular window is computed using Eq. 3 (see “Methods”). **B** Scatterplots showing predictive power (correlation) between $p_{new}$ and the fraction of undetected SV for a 10kbp and 100kbp window and two sample sizes 100 genomes (top panels) and 1000 genomes (bottom panels), sub-sampled from the 1KGP data. The x-axis shows the prediction made by SVhound (probability of new SV-allele, $p_{new}$) and the y-axis shows the proportion of undetected SV-alleles in the non-sampled individuals ($f_{undetected}$). Be aware that the axis ranges have been adopted to better visualize the results. Note that regardless of sample size, SVhound performs better in the 100kbp window when comparing both window lengths. **C** Distribution of the probabilities of detecting a clSV ($p_{new}$) for different window lengths

Figure 1A exemplifies this for three windows and a sample of *n* = 100 genomes. In windows *w*_*1*_*, w*_*2*_*, w*_*3*_, we detected *k* = 3, 5, 2 SV-alleles, leading to diversity parameter estimates $\theta \left( {w_{1} } \right) = 0.430, \theta \left( {w_{2} } \right) = 0.948, \theta \left( {w_{3} } \right) = 0.204$ and the probabilities to find a clSV in the respective windows, if an additional genome or sequence from the respective window is sequenced, equal $p_{new} \left( {w_{1} } \right) = 0.00430,p_{new} \left( {w_{2} } \right) = 0.009390, p_{new} \left( {w_{3} } \right) = 0.00205.$

To investigate the power of SVhound to predict clSVs and to study the influences of the window-length and sample size, we randomly sub-sampled 50 (2.00%), 100 (4.00%), 500 (19.97%) and 1000 (39.34%) human genomes from the 2504 genomes of the 1KGP [20] for a variety of window lengths (5, 10, 50, 100, 200, 500 and 1000 kbp). For each of the 28 combinations of window lengths and sample sizes we compared the $p_{new}$ estimates with the fraction $f_{undetected}$ of SV-alleles that do not occur in the respective sub-sample, but are present in the full 1KGP data (see “Methods”).

Figures 1B displays the association between $p_{new}$ and $f_{undetected}$ for sub-samples of size n = 100 (Fig. 1B top panel) and n = 1000 genomes (Fig. 1B bottom panel) and window lengths of 10kbp and 100kbp, respectively. We observed that the window length had a bigger impact on the performance of SVhound by evaluating $p_{new}$; for example the correlation coefficient (r) for 10kbp window is r = 0.3976 and r = 0.1698 for 100 and 1000 genomes respectively (Fig. 1B top panel), while for 100kbp window the performance of SVhound greatly improves with r = 0.8519 for 100 genomes and r = 0.9524 for 1000. We also noticed that the sample size only improved the correlation coefficient for window lengths of at least 50kbp. The scatterplots of the 28 window-sample size combinations are shown in Additional file 1: Fig. S1.

While the above analysis was based on one simulation, we performed 100 simulations for each of the 28 parameter combinations. Additional file 1: Fig. S2 and Additional file 1: Fig. S3 show the distribution of the correlation coefficients, the coefficients of determination ($r^{2}$) and the slopes for the 100 simulations and the observations exemplified in Fig. 1B are corroborated.

Additional file 6: Table S1.1 shows the average correlation coefficients for the 100 simulations for each of the 28 window-sample size combinations. If the window length is large and the sample size is large then we observe a high correlation between $p_{new}$ and $f_{undetected}$, with large window lengths we have more data to estimate the model parameter and thus the predictions improve. But not only the correlation is high for large windows, also the slope of the regression line approaches one with increasing sample size and window length (Additional file 6: Table S1.2). This indicates that $p_{new}$ is indeed a good predictor of $f_{undetected} .$

We note that, with increasing window length $p_{new}$ increases (see also Fig. 1C), which can be explained with the infinite allele assumption almost being met and thus the probability to find clSVs increases. Contrary, the increase in sample size has the opposite effect (Additional file 1: Fig. S4). With increasing window length the chances also increase to find SV-alleles that occur exactly once, high numbers of such singletons will increase the diversity parameter, $\theta ,$ and subsequently $p_{new}$ (see “Methods”). However, with larger window lengths the resolution and thus the genomic location of the predicted additional SV-alleles is reduced.

We further investigate what drives the increase in predictiveness with the increase of window length. We note that for small window lengths the average number of SV-alleles per window was low and thus affects the diversity-parameter estimation. Additional file 1: Fig. S1 shows that 100kbp was the smallest window length were an increase in sample size improves the correlation ($r^{2}$) and the slope approaches one. We computed the average number of SV-alleles for the 100kbp window using the whole 1KGP dataset and found that genome-wide we have on average 10 SV-alleles per window, which we use in all following analyses to estimate the appropriate window size to each dataset.

### Identification of polymorphic candidate regions across 2504 human genomes from the 1000 genome project

**Fig. 2:**
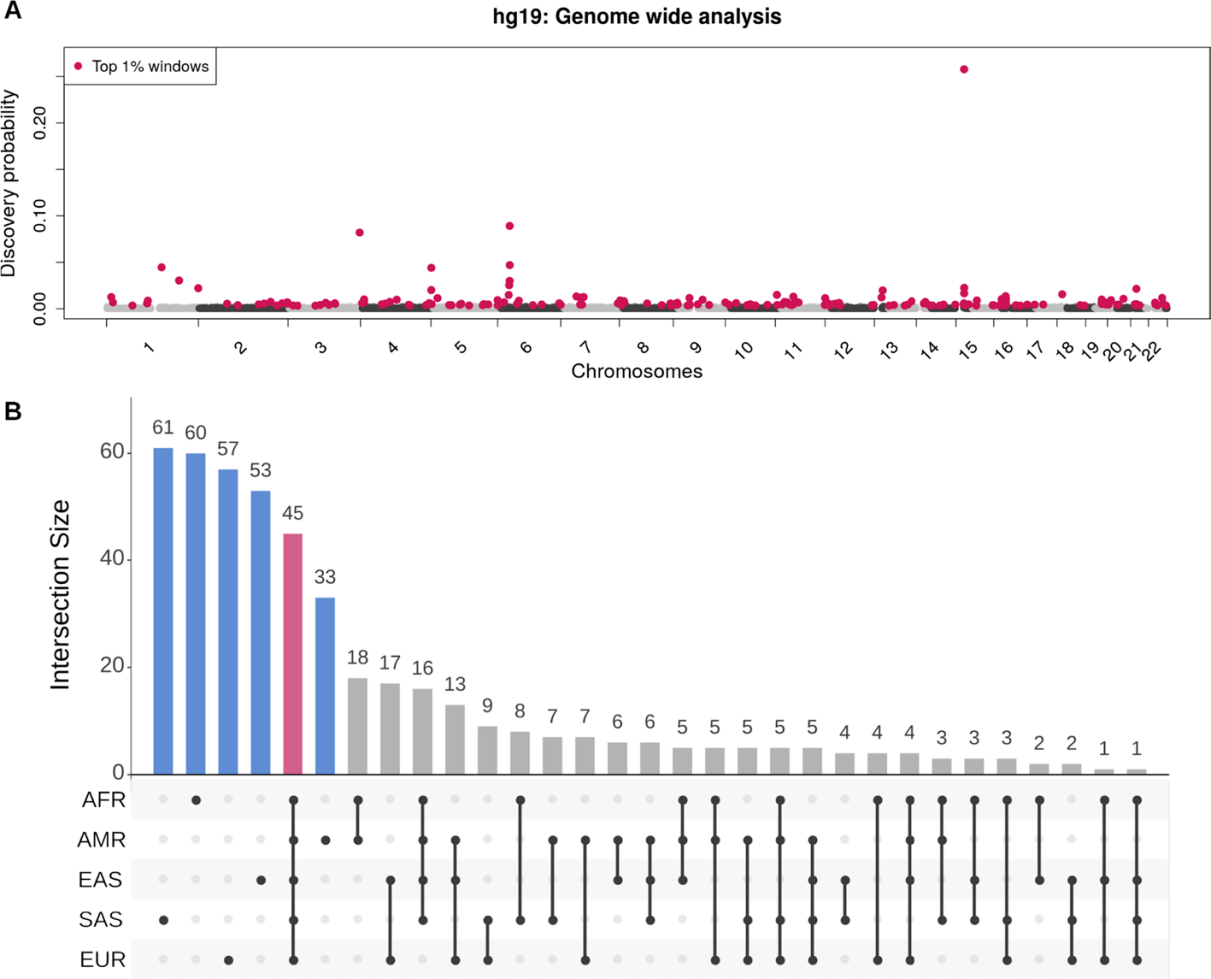
**A** Genome wide distribution of $p_{new}$ for the 2504 genomes (100 kbp window) from the 1KGP data set. Red dots show the 188 candidate windows ($p_{new} \ge 0.34\%$) along the 22 human autosomes (hg19), gray/black (alternating shades by chromosome) dots display the $p_{new}$ for the remaining windows. Note the window on chromosome 15 with a $p_{new} = 25.77\%$, contains two olfactory receptor proteins, four olfactory receptor pseudogenes, multiple CNVs and an LINE1 insertion. **B** Distribution of 468 candidate windows when decomposing the 1KGP data set into the five super-population: African, AFR; Admixed American, AMR; European, EUR; East Asian EAS; South Asian, SAS. The black dots below each bar display the occurrences of the candidate windows in each population. Ancestry specific windows, i.e. present in one population are blue, ubiquitous windows are red

We applied SVhound to the 2504 genomes of 1KGP SV calls to identify likely regions (loci) with clSV. SVhound automatically calibrated the window length to 100kbp. The human genome was then partitioned into 18,397 windows from which we analyze the top candidate loci, representing 1% of the windows with the highest probability of detecting clSVs ($p_{new} \ge 0.34\%$). Figure 2A shows the probabilities of detecting a clSV for each window. The red dots mark the top 1% (188) windows with the highest $p_{new}$ for clSVs (here thereafter candidate windows). The most noticeable candidate window is located on chromosome 15 with $p_{new}$ = 25.77% of detecting a clSV if one new sample is added. The remaining windows with $p_{new} < 0.34\%$ are not considered in the analysis (in dark/light gray).

We are particularly interested where in the human genome the 188 candidate windows occur. To achieve this, we overlapped the candidate windows to several annotation databases. First, we investigate whether these candidate windows are identified only in intergenic regions or if these windows are actually preferentially located near genes. As windows are large enough, each window can overlap with more than one annotated element. We found 107 candidate windows that overlap with 204 protein coding genes (Additional file 6: Table S2), 148 candidate windows overlapping non coding genetic elements (Additional file 1: Fig. S5) and 24 windows in intergenic regions. Next, to understand the biological role of the 204 genes we performed an enrichment analysis with PANTHER [21], and found enrichment for biological processes related to: cellular detoxification of nitrogen compound, xenobiotic catabolic process, interferon-gamma-mediated signaling pathway, regulation of immune response and sensory perception of smell (Additional file 6: Table S2 and Additional file 6: Table S3). The noticeable candidate window that we observed on chromosome 15 contains two olfactory receptor proteins and four olfactory receptor pseudogenes (Fig. 2A).

Next, we investigate whether SVhound is suggesting regions containing repeats that are known to show many structural variants [9]. For this we analyzed the overlap of the candidate windows with annotated repeat elements from the RepeatMasker track [22], simple tandem repeat elements [23] and segmental duplications [24]. First, we found that the LINE and LTR repeat families were the most often observed in candidate windows, with the L1-LINE repeat [23] being the most abundant, followed by LTR-ERV1 (Additional file 6: Table S4.1 and Additional file 6: Table S4.2). Next, for the case of simple tandem repeat elements, we found all but one candidate window overlapped with at least one of these elements, which coincides with their abundance in the human genome. We observed that these ubiquitous elements were not present more abundantly within the candidate windows when compared to the rest of the genome (T-test of difference in means p-value = 0.314, Kolmogorov–Smirnov test p-value = 0.2378, Additional file 1: Fig. S6). Finally, for the segmental duplications [24] we found 101 candidate windows overlap with at least one segmental duplication, from which 88 overlapped with multiple ones (Additional file 6: Table S5).

Next, we wondered if SVhound actually only identifies repeats or indeed regions that will harbor undetected SV. Low complexity repeats, for example, are often the cause of falsely identified SV and thus maybe do not always harbor these clSV. To assess this we focused on non repetitive regions such as the high confidence regions defined by the Genome in a Bottle Consortium (GIAB, [16, 25]) representing reliable regions for structural variation detections using short reads (e.g. outside of segmental duplications, low mapping quality regions) and thus potential targets for experimental validation. We found that only 18 out of the 188 candidate windows (9.57%) did not overlap with the high confidence regions annotated by the GIAB Consortium [16] (Additional file 6: Table S6). Therefore, SVhound indeed reports loci with biological significance rather than enriching for artifacts or regions known to be variable in the genome (e.g. intergenic). Finally, we compared the results of SVhound to two different approaches of investigating SV in a population: (1) a classic approach of detecting SV hotspots in the genome and (2) a comparison to rare alleles (MAF < 1%, see “Methods”).

For the first case, we used the hotspot analysis of Lin and Gokcumen [26], which divided the genome in 100 kb windows and then we used the same coordinates to identify candidate windows with SVhound. We found that 83 windows were considered both a hotspot and a candidate window by SVhound (34.6%, Additional file 1: Fig. S7). Moreover, 157 (65.4%) of the candidate windows were not cataloged hotspots, thus showing that SVhound detects both hotspots and non-hotspots as candidates for further analysis. This result is not surprising, because SVhound computes the probability to find a new SV. This probability depends on the number of SVs in the window and the sample size (see “Methods”) in a non-linear way. For the second approach, we performed a comparison between rare observed SVs (low frequency SV, MAF < 1%) and the candidate windows proposed by SVhound. We found 22,386 SV that fall in the category of having “rare alleles”, from which only 967 of such “rare alleles” overlapped with a candidate window. These results clearly indicate a difference between the results one can expect from these two approaches when compared to SVhound.

Next, we applied SVhound to identify SV confined to particular human ancestries defined in the 1000 genomes project (African (AFR), Admixed American (AMR), European (EUR), East Asian (EAS) and South Asian (SAS)). We split the 2504 genomes into five so-called “super-populations” (661 AFR, 347 AMR, 503 EUR, 504 EAS, 489 SAS) and scanned for candidate windows by repeating the previous analysis for each ancestry. Additional file 1: Fig. S8 shows the candidate windows (top 1% with highest $p_{new}$) for each of the five studied populations. From the collection of all top 1% candidate windows (total number of distinct windows: 468) we investigated those present in a single population (ancestry-specific windows) and thus identified potential regions of high polymorphism specific to a particular population; and those that occurred in all populations (ubiquitous windows) and thus represent regions of high polymorphism in the all humankind (Fig. 2B, Additional file 6: Table S7).

We detected 45 (9.62%) ubiquitous windows, whereas 264 (56.41%) windows were ancestry-specific, which break down as follows: South Asian, 61; African, 60; European, 57; East Asian, 53; Admixed American, 33. Finally, the remaining 159 (33.97%) candidate windows occurred in two to four populations.

Next, we investigated the role of the genes in the ubiquitous and the ancestry-specific windows (Additional file 6: Table S8). For the genes in the ubiquitous windows, we found enrichment in biological processes also found in the 1KGP full data set (nitrobenzene metabolic process, cellular detoxification of nitrogen compound, xenobiotic catabolic process, interferon-gamma-mediated signaling pathway, antigen processing) (Additional file 6: Table S9.1). When analyzing the ancestry-specific windows, we only found gene enrichment in the South Asian population for 8 biological processes related to keratinization (tissue development, Additional file 6: Table S9.6).

Finally, we analyzed if repeat elements overlap with ubiquitous and ethnic specific candidate windows. Here, the L1 (LINE), ERV1 (LTR) and ERVL-MaLR (LTR) repeats were the most abundant among both ubiquitous and ancestry specific candidate windows (Additional file 6: Table S10.1). Next, when analyzing the repeat elements present in a single ethnic group, LTR Gypsy-like is an example that overlaps with the ancestry specific windows of the African population [27]. Similarly, an ERVL-like (LRT) repeat is restricted to ancestry specific windows for European population, the TcMar-Tc2 (DNA repeat) was found in ancestry specific windows for the Admixed American population and Satellite-telo in the South Asian population (Additional file 6: Table S10.2).

### Identification of polymorphic candidate regions across 19,652 human genomes in the USA

**Fig. 3:**
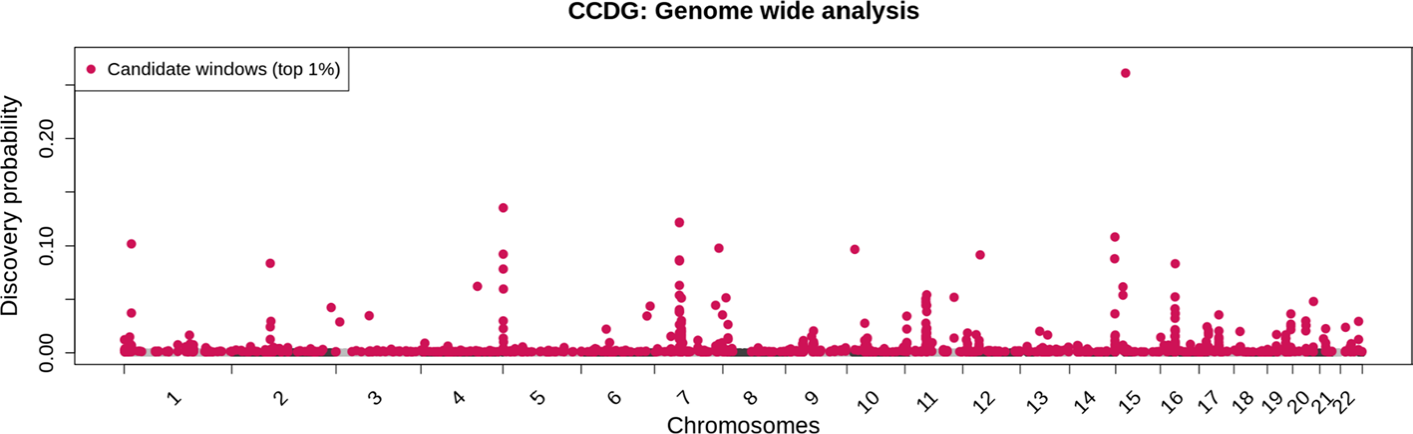
Genome wide analysis of the CCDG data set. Red dots display the top 1% candidate windows (1282) along the 22 autosomes of the human genome (hg38)

To extend our work further, we applied SVhound to detect regions with undetected SVs in 19,652 genomes of US residents (CCDG data) that include 8969 European-American, 8099 Hispanic or Latino-American and 2584 African-American genomes [4]. SVhound automatically estimated the optimal window length to be 10kbp. We again considered as candidate windows those representing 1% with the highest probability of detecting a clSV ($p_{new} \ge 0.081\%$). Figure 3 shows the distribution of the probabilities to detect a clSV when splitting the genomes in 126,185 windows, highlighting in red the 1282 the candidate windows.

Next, we used a similar annotation strategy to the 1KGP over the 1282 candidate windows, overlapping them to several databases. We found 381 candidate windows that overlapped with 331 protein coding genes (Additional file 6: Table S11), 396 overlapping non coding genetic elements (Additional file 1: Fig. S9) and 599 windows in intergenic regions. Again, we performed an enrichment analysis with PANTHER using the 331 genes and found gene enrichment for 27 biological processes, all of them related to immune response, e.g. phagocytosis, homophilic cell adhesion via plasma membrane adhesion molecules, complement activation, B cell receptor signaling pathway, positive regulation of B cell activation among others (Additional file 6: Table S12).

Next, we analyzed the repeat elements that lay within the candidate windows (Additional file 6: Table S13.1 and Additional file 6: Table S13.2) and observed an overall increase in the number of repeats overlapping with candidate windows. The LINE and LTR families were found in 44.2% and 30.66% of the candidate windows, which represent a decrease of 20.67% for the LINE and 23.6% for the LTR when compared to the 1KGP data. In addition, the DNA repeats were found in 23.79%% of the candidate windows, while the rest of repeat elements are found in less than 3% of the windows.

Next, we analyzed the presence of simple tandem repeats within the candidate windows of the CCDG dataset. Here, we found significant differences in the average number and the distribution of simple tandem repeats across the 1282 candidate windows (T-test p-value < 2.2e−16, Kolmogorov–Smirnov test p-value = 1.453e−08, Additional file 1: Fig. S10), result that deviates again from our analysis of 1KGP data. We found that the candidate windows from the CCDG dataset overlapped with centromeric and pericentromeric regions, which tend to be abundant in highly repetitive sequences [28] and repeat elements and were likely inaccessible/filtered from the 1KGP dataset.

Finally, we noticed consecutive clusters of candidate windows (ten or more consecutive windows cataloged as candidates) along some genomic regions (Additional file 6: Table S14)*.* We found such clusters of candidate windows in chromosomes 5 (two clusters of size 12), 7 (three cluster sizes 17, 16 and 28), 9 (cluster size 12), 11 (cluster sizes 12 and 13), 12 (cluster size 12), 14 (cluster sizes 11 and 10), 17 (cluster size 16), and 19 (cluster size 25). One cluster is located near the telomere (chromosome 5) and seven in pericentromeric regions (chromosomes 5, 7, 11, 12) which are well known for having a high density of simple repeats, satellite repeats, and repeat elements in general (LINE, LTR, etc.) and coincide with the instability of such regions in genome assemblies, which are known to be hard to resolve due to their repetitive nature.

Further, five clusters are within coding regions in chromosomes 9, 14, 17 and 19. Here, it is prominent the case of a 155kbp region in chromosome 9 that overlaps with a novel lncRNA (ENSG00000285784). Next, we found a 169kbp region on chromosome 14 that include eight olfactory receptor genes, a 123kbp region on chromosome 14 which include 4 immunoglobulin genes (IGHA2, IGHE, IGHG4, IGHG2) two miRNA (MIR8071-1, MIR8071-2) and a lncRNA (COPDA1), a 185kbp region in chromosome 17 which include the KAT8 regulatory NSL complex (KANSL1, also observed in the GWAS analysis) and a 301 kbp region in chromosome 19 where we found six pregnancy specific beta-1-glycoprotein and two lncRNA (PSG8-AS1, ENSG00000282943). Thus, many of these clusters of candidate genomic regions are already well known to be highly variable.

We then focused on segmental duplications overlapping candidate windows. Here, we observed a slight decrease in the number of candidate windows overlapping with a segmental duplication (41.5%) when compared to the 1KGP (53.7%) (Additional file 6: Table S15). We identified the candidate windows that overlapped with the GIAB high confidence regions that exclude regions where short reads cannot reliably identify SV. Overall, 69.4% (890) of candidate windows overlapped with these “high-confidence” regions and thus indicate that reliable SV calling can be achieved in such regions [16]. (Additional file 6: Table S16).

Finally, we compared the results of the two independent human datasets, (1KGP, CCDG) that we analyzed with SVhound to examine the similarities in the prediction. As each dataset was analyzed with distinct genome reference, we compared the shared genes that overlapped with candidate windows. Surprisingly, we found only 41 genes present in candidate windows of both the 1KGP and CCDG data sets, representing approx 8.3% of the 495 genes associated with at least one of the candidate windows from the 1KGP or CCDG data (Additional file 6: Table S17). This small intersection may be related to the fact that the CCDG dataset focuses on the US population while the 1KGP dataset comprises 26 different ethnicities [20], coupled with the difference in number of candidate windows (188 in the 1KGP dataset to 1282 in the CCDG dataset, see Additional file 6: Table S18.1).

### Identification of SV and further polymorphic candidate regions across 150 Rhesus Macaques

**Fig. 4:**
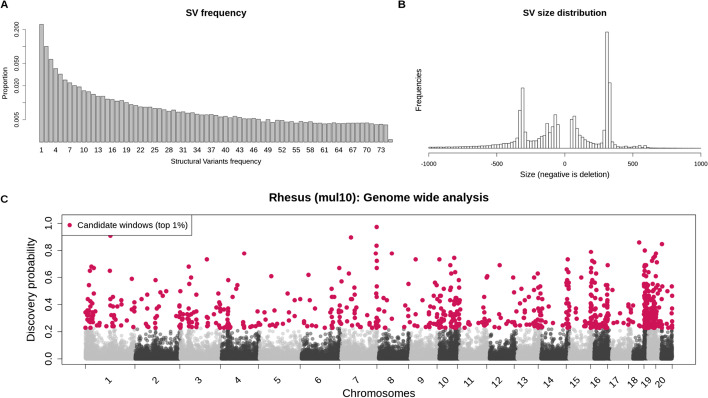
**A** Logarithmic scale of allele frequency distribution of the SV called in 150 rhesus macaque genomes for all SV types. **B** length distribution of the insertions (positive) and deletions (negative) called in the rhesus macaque genome (truncated at ± 1000 bp, see the full binned table in Additional file 6: Table S19). **C** Genome wide analysis of the rhesus macaque (Macaca mulatta, Mmul_8) data set. In red are shown the 1101 candidate windows ($p_{new} \ge 22.3\%$) along the 20 autosomes of the macaque genome, in gray (alternating shades by chromosome) are shown the rest of the windows

Finally, we applied SVhound to 150 whole genome sequences from the rhesus macaque (*Macaca mulatta*), a widely used primate model of human disease that has not been well studied with respect to SV [18, 19]. For this we created a novel catalog of SV for rhesus macaques by comparing 150 genomes to the reference Mmul_8 (see “Methods”, [8]). We identified SVs among the genomes of these 150 rhesus macaques that came from several US research colonies (see “Methods” for details). The largest proportion of SVs were deletions (45.84%) followed by insertions (36.88%), inversions (11.45%) and tandem duplications (5.82%) (Additional file 6: Table S19.1 and Additional file 6: Table S19.2). This follows roughly the distribution expected from human SV datasets [9]. Interestingly, we found a high number of SVs on chromosome 19 (Additional file 6: Table S19.3). Chromosome 19 includes tandem repeats of olfactory receptors, KIR (killer cell immunoglobulin-like receptor) loci and other immunology genes and was previously shown to have a higher rate of both CNV and SNV polymorphism than other macaque chromosomes [18, 29]. Figure 4A shows the minor allele frequency (MAF) spectrum. The MAF spectrum for the genome wide SVs follows the same distributions as in other populations (e.g. human), with the majority of the 102,572 SVs (53.7%) exhibiting low frequency (MAF < 0.05). We observe 5946 SV having an MAF > 45%, which might be because the reference genome contains an array of low frequency SVs. Interestingly, we noticed a profound peak for Alu insertions (Fig. 4B) that highlights Alu activity in this species.

We applied SVhound to identify candidate regions that may contain undiscovered variation. First, we observed that the rhesus raw data contained a larger number of SVs when compared to the human dataset (Additional file 6: Table S18.1), even though the number of genomes was an order of magnitude smaller when compared to the 1KGP and two orders of magnitude smaller when compared to the CCDG. This time SVhound estimated a window length of 27kbp. Here, given the small sample size, the non candidate windows presented higher $p_{new}$ discovery probabilities when compared to the two full human datasets and similar to those in the subsamples (e.g. 100 individuales of 1KPG in Fig. 1B, top panels).

We extracted the top 1% candidate windows from the 75,554 windows $(p_{new} \ge 22.3\%$, Fig. 4C). Then, we extracted 479 annotated rhesus genes that overlap with a candidate window and performed an enrichment analysis with PANTHER (unmapped ID not counted, Additional file 6: Table S20). We did not find any significant enrichment for biological processes (Additional file 6: Table S21) probably also because of the small sample size.

### Utilization of SVhound for quality control (QC) of population studies

Given SVhounds ability to automatically adjust and determine regions of clSV, we next investigated if it can also be leveraged to QC population SV data sets. By utilizing the SV-density coupled with the number of different SV-alleles, $k$, one can assess the quality of a given dataset. As an example, we compare a subset of 150 genomes from the 1KGP and the rhesus dataset (also 150 genomes). Even when both datasets have the same sample size, the window length selected for rhesus is 27kbp, while for the 1KGP dataset is 319kbp (Additional file 6: Table S18.2).

First, we noticed that the distribution of the $p_{new}$ values is similar with an average $p_{new} = 1.85\%$ for the 1KGP and $p_{new} = 2\%$ for the rhesus dataset (median $p_{new} = 0.9\%$, $p_{new} = 0.73\%$, and max $p_{new} = 94.7\%$ and $p_{new} = 97.3\%$ respectively) which show consistency on the $p_{new}$ values, regardless of the dataset, when the desired SV-density remains the same.

**Fig. 5:**
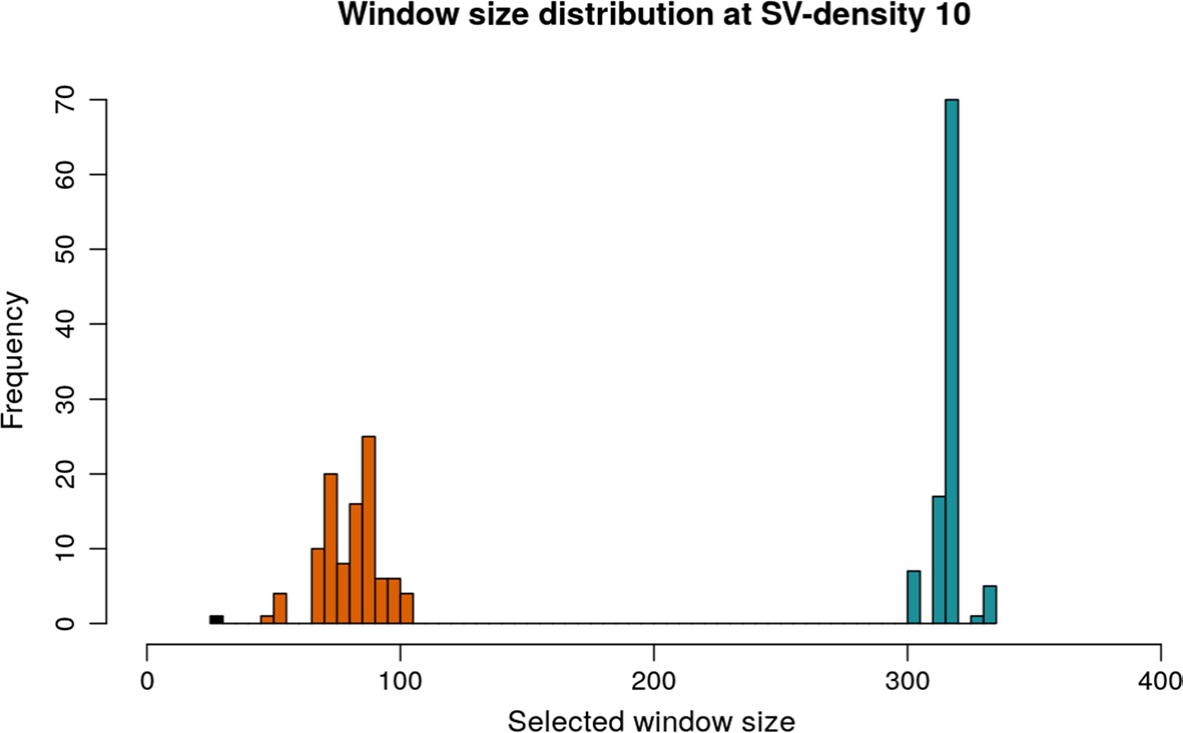
Window length selection for the 1KGP, CCDG and rhesus datasets. Random samples of 150 individuals were taken from the 1KGP and CCDG datasets and window length selection was calculated for an average SV-density of 10 SV per window. In black (furthest left) is the rhesus data with a selected window length of 27kbp that represents the noisiest dataset. Next, we see in orange the distribution for the CCDG data and finally, in blue for 1KGP (most curated)

Next, we included in the analysis a total of 100 random samples of 150 individuals from the 1KGP and 100 random samples of 150 individuals from the CCDG. We observe that for each dataset, the selected window length lies in its own distribution (Fig. 5). These window lengths reflect two important aspects of the dataset: first, the overall number of SV in each particular dataset, with 1KGP having 66,626, the CCDG dataset 304,533 and the rhesus 493,188 (Additional file 6: Table S18.1). When randomly removing SVs from the CCDG dataset, we observe an increase in the window length (Additional file 2: Fig. S11). This is also observed in the 1KGP dataset. Second, given a fixed SV-density, the distribution of the number of different SV-alleles, $k$, reflects a similar distribution despite the difference in window length. This distribution mimics the allele frequency spectrum, where most windows have few SV-alleles and only a small number of windows (the candidate ones) have a high number of SV-alleles (Additional file 3: Fig. S12, Additional file 4: Fig. S13, Additional file 5: Fig. S14). We can use this expected distribution of $k$ to detect possible errors and biases in the data that can be caused by a defined population structure, an increase in the number of falsely called SV or possible contamination. Given these insights, SVhound indeed can be utilized also to QC SV population catalogs and will identify a deviation from the expected “L” shaped distribution of the number of different SV-alleles, $k$. Finally, we can examine the probability of detecting new SV-alleles to identify saturation and focus the efforts in specific genomic loci or new species.

## Discussion

We developed SVhound to investigate regions along the genome that are likely to harbor SV across yet unsequenced samples (clairvoyant SV or clSV), exemplifying the method with an analysis of human and rhesus genomes. We demonstrate that SVhound finds regions of undetected variation that harbor genes and are not simply enriched for repeats or intergenic regions along the genome. Moreover, many of these regions are accessible by short reads, which would allow the design of a targeted strategy to sequence these regions with both short and long read technology [30]. This undetected variation indicates the likely importance of such regions where we are missing alleles that may have an impact in evolution and medicine, which may contribute to missing heritability [31]. Nevertheless, future studies will need to conduct broader investigations if these clSV candidate windows represent further signals of evolution or other impact across these populations. SVhound utilizes a sampling scheme approach derived from population genetics (Ewens 1972) to model the SV-allele distribution and to predict genomic regions with high probability of clSV. SVhound showed a high accuracy over the 1KGP data when assessing its prediction power with a high correlation coefficient across multiple parameters (median correlation across 24 tested parameters = 0.913, best r = 0.993) and slopes close to 1. Apart from the obvious observation that increasing the window length would increase the probability of detecting a clSV (for a 100Mbp window length of course there will be a clSV), we found that the lack of SV in windows (e.g. very low SV-density) lead to imprecise predictions, likely due to violations of the model assumptions. Across the 1KGP datasets, the method performed well for an SV-density = 10, which corresponds to 100kbp windows (average correlation of 0.894 of 400 evaluations) and even better when considering SV-densities ≥ 10 (window lengths 200kbp, 500kbp, 1Mbp) where the average correlation was > 0.95 for more than half the evaluations (min. correlation = 0.8189). Remarkably the prediction to find clSVs is sample dependent. The CCDG data with a large sample of 19,000 human genomes exhibited smaller ${p}_{new}$ values compared to 1KGP (Additional file 6: Table S22.2). This difference is resolved when the data processing of each dataset is taken into account. For the CCDG dataset 304,533 SVs were determined, compared to 66,626 SVs for the 1KGP. This difference might reflect the way SVs were called in the 1KGP project, where the majority of genomes had low coverage (3-5x) and likely suffered from a low SV sensitivity, thus leading to an underestimation of the general variability. A conservative SV-calling approach will lead to an underestimation of $\theta$ and thus the probability to detect new SVs is also reduced.

The SV-calling procedure in the CCDG project used genomes with a much higher read coverage, thus had more power to detect SVs. These two data sets are hard therefore to compare and clearly shows that SVhound accuracy also relies on the experimental design of the underlying data. The difference might be reduced in the recently posted 1KGP data set where all samples had ~ 30 × coverage [32]. For rhesus macaques we used the same strategy of utilizing the SV-density as the driving factor to determine the window length. Even when we had a smaller cohort (only 150 genomes), a high number of SVs were identified (493,188 SVs), with a different composition (e.g. we identified an abundance of SV especially insertions).

SVhound successfully identified for all three genome projects (1KGP, CCDG, rhesus) genomic regions with a substantial probability to harbor clSVs. It is noteworthy that SVhound does not require any other annotations than SV coordinates in a region. Several candidate regions were confined to well-known regions of high genomic diversity like immune regulatory genes for antigen processing and antigen binding genes (HLA), olfactory genes, regions overlapping repeat elements (LINE, LTR) and regions with an overrepresentation of simple repeat elements (telomeric and pericentromeric regions). Moreover, we identified other genomic loci with high probabilities of harboring new SV-alleles that contained for example a pregnancy specific beta-1-glycoprotein and novel lncRNA genes.

It is of course not only interesting which regions SVhound predicts will likely harbor additional not yet observed SV. Thus the question is also what these regions represent. It is clear from our analysis that regions with a high probability of clSV represent areas that have not completely been characterized nor sampled. Thus including additional mutations with potentially high impact as shown with overlaps with immune related loci/genes. The regions identified here might also correlate with increased instability in the genome. We have tested here the correlation with repeats directly and did not identify a significant correlation. We can also ask what are the implications? After sequencing hundreds of thousands of genomes, the question might arise whether whole genome sequencing is indeed the most efficient strategy to obtain a more complete set of variations within a particular population of a species. An alternative strategy would be to use a capture (e.g. Cas9 [30] or selective sequencing [33]) design to investigate the identified regions that provide the largest likelihood of containing additional SV-alleles. However, it remains challenging to design these panels for certain regions (e.g. MHC). Nevertheless, it would indeed represent a more efficient strategy to design capture reagents for certain regions and use them to perform targeted sequencing in additional samples to improve the catalog of human population variations. The obvious downside of such an approach is of course that we would likely miss other (rare) SV-alleles in the regions outside of these panels and we don't know yet if SNV would follow the same trend that we observed for SV. Thus, the challenge remains to obtain a full catalog of common variations across the human population, and also for other important research species.

SVhound can assist with prioritizing regions independently of the organism that is being studied (e.g. non model organism). In addition, SVhound can also indicate that a given population is under-investigated for SV (e.g. rhesus data in this manuscript). While this may be obvious given our sample size of 150, we observe it not so obvious in the 1KGP for the same sample size, and it might not be as obvious in many cases, when the sample size reaches thousands. Here SVhound can again assist in estimating the quality of an SV call set for a given population by means of its estimated window length. Datasets that are excessively curated, or present bias towards certain genotypes will present large window lengths, while too noisy datasets will present smaller ones.

## Conclusions

SVhound shows high prediction accuracy for highlighting regions of the genome where additional SV should be found. Such regions are not only present in well known variable regions (e.g. centromere, HLA-locus) and can help scientists to focus their efforts in understudied regions. This can be resolved either via additional sequencing or improved analysis methods across the data sets in these regions.

## Methods

### Summarization of the structural variants (SV)

We study the genomic variation of a sample of completely sequenced individuals in disjoint fixed windows and analyze each window as follows.

To simplify wording, think of a window as a locus, then each distinct SV (particular set of SV present in a given window) is considered as SV-allele. For a sample of *n* individuals from this window, we count how often individuals with exactly the same SV in the window occur. With $a_{i}$ we count the number of different SV-alleles, that occur exactly $i$-times, where $\mathop \sum \nolimits_{i = 1}^{n} ia_{i} = n.$ We call $a = \left( {a_{1} ,a_{2} , \ldots ,a_{n} } \right)$ SV-occupancy vector. $a_{1}$ describes the number of different SV-alleles each occurring exactly once in the sample. If $a_{n} = n$, then all individuals carry the same SV-allele in the window. Finally $\mathop \sum \nolimits_{i = 1}^{n} a_{i} = k$ describes the number of different SV-alleles in the window.

We notice that the SV-*occupancy vector* assumes the role of the allele frequency spectrum (AFS) in population genetics [34]. However, the AFS is computed for alleles from a gene, whereas the *SV-occupany vector* is computed from the different SV-alleles in a window. Since the potential number of SV-alleles in a *large enough* window is big, the infinite allele assumption is not severely violated and the well known Ewens Sampling Formula [34] that describes the probability to observe a SV-occupancy vector:

1 $$\Pr (a_{1} , \ldots ,a_{n} ;\theta ) = \frac{n!}{{\theta (\theta + 1) \ldots (\theta + n - 1)}}\prod\limits_{j = 1}^{n} {\frac{{\theta^{{a_{j} }} }}{{j^{{a_{j} }} a_{j} !}},}$$

holds, where *θ* is a measure for the genetic diversity of the population. Although Ewens (1972) developed the theory to understand the sampling theory of neutral alleles, we note that the EWS is relevant in very diverse scientific disciplines (see: Harry Crane (2016) The ubiquitous Ewens sampling formula. Statistical Science 31:1–19). Equation (1) and the SV-occupancy vector can be used to compute a maximum likelihood estimator for $\theta$, since this is numerically challenging, we used a simpler approach.

To estimate parameter *θ* based on a sample of *n* individuals, it suffices to apply the method of moment by replacing $E\left( K \right)$, the expected number of SV-alleles by the observed number of alleles $k$ and then numerically solve the next equation

2 $$E\left( K \right) = \frac{\theta }{\theta } + \frac{\theta }{\theta + 1} + ... + \frac{\theta }{\theta + n - 1}$$

for $\theta$. In fact, $\hat{\theta }$ is the maximum likelihood estimate for the data.

Having an estimate, $\hat{\theta },$ we use this value to compute the “predictive” probability to find a clairvoyant SV (clSV, defined as a new or previously undetected SV-allele of unknown genotype) if a new window from an individual is sequenced as:

3 $$p_{new} = \frac{{\hat{\theta }}}{{\hat{\theta } + n}},$$

Equation 18 in Ewens [34].

Please, note that if $\theta$ is small we expect a small number of SV-alleles, a large $\theta$ implies that each SV-allele occurs once. However, for such cases to occur $\theta$ must be extremely small/large. Finally, notice that $p_{new } = 0$ if a window has the same sequence across the entire sample (k = 1).

To validate SVhound, we partitioned the human genome in non-overlapping windows of size 5, 10, 50, 100, 200, 500, and 1000 kbp. For each window, we randomly re-sampled $n = 50, 100, 500, 1000$ individuals without replacement from the 2504 individuals in the 1000 human genome project [20] version hg19. This re-sampling was repeated 100 times.

For each subsample, we estimated $\hat{\theta }$ from Eq. (2) and then estimated the probability to find a clSV, $p_{new}$, based on Eq. (3). $p_{new}$ was subsequently compared to the proportion of individuals that were not in the subsample and that carried SV-alleles not yet detected, $f_{undetected}$ that is we computed.

4 $$f_{undetected} = \frac{\#\, of\,individuals\,with\,SV - alleles\,not\,in\,the\,subsample}{{\#\,individuals\,not\,in\,subsample}}$$

### Automatic window length selection

Based on the evaluation of the window and sample sizes described above in “validation of SVhound”; we opted automatically select the window size, such that we select the shortest window length with enough information to accurately estimate the model parameter $\hat{\theta }$. During the validation we observed that 100kbp was the point where increasing the sample size, greatly improved the SVhound prediction ($p_{new}$). Next, we computed the genome-wide average number of SV when using the 100kbp window length in the 1KGP dataset (SV-density). We identified that for this window size (100kbp), the SV-density is 10, meaning on average a window has 10 SV-alleles. We then used the SV-density of 10 to compute the appropriate window length in the rest of the paper. First, we start at 10kbp window size and use the first 1000 SV from the VCF file to compute the SV-density. Then, if the SV-density is not close to 10 (10 ± e, with e = 0.2 by default but can be user defined), we adjust the window size using a bisection method, with a lower bound of 10kbp and an upper bound of 1Mbp.

### Identifying SV variability in the human genomes

We performed a genome-wide analysis to identify genomic regions with a high probability of harboring new SV-alleles. We used two human datasets: a sample of 2504 individuals for the case of the 1KGP dataset and 19,652 individuals from the Centers for Common Disease Genomics project dataset [4]. For both datasets we estimated the window length for a SV-density of 10. The estimated window length for the 1KGP dataset was 100kbp and 10kbp for the CCDG dataset. We used the script vcf_autoparser_for_svhound.py (see Data access) to parse the VCF files into a tab-separated table input of SVhound. We then estimated the diversity parameter $\hat{\theta }$ for each window using Eq. 2 to then calculate the probability of observing a new allele in the next individual using Eq. 3. We selected candidate windows as the 1% windows with the highest probability of detecting a clSV in the next sequenced individual $(p_{new} )$. From these regions we extracted genomic features information from the proper annotation of the human genome [22, 35, 36] (depending on the reference used) to detect what type of genetic elements may be affected.

We performed the enrichment analysis with PANTHER [21]. We also used data of the position of repeat elements, simple tandem repeats [23], segmental duplications [24],reference “high-confidence” regions from the GIAB project [16, 25] and SNP information from the GWAS catalog [37].

### Identifying SV variability in the macaque genomes

We performed a genome-wide analysis to identify genomic regions with a high probability of harboring new SV-alleles. We used a rhesus macaque dataset composed of 150 genomes, for which we estimated the window length as with the human datasets (genome-wide average SV-density = 10). We used a window length of 27 kbp and used the script vcf_autoparser_for_svhound.py to parse the VCF files into a tab-separated table input of SVhound. Then we estimated $\hat{\theta }$ for each window using Eq. 2 to then calculate the probability of observing a new allele in the next individual using Eq. 3.

Then, we selected candidate windows as the 1% windows with the highest probability of detecting a clSV in the next sequenced individual ($p_{new}$). From these regions we extracted genomic features information from the rhesus macaque genome annotation from Ensembl release 97 (Mmul_8) [38].

### Annotation for the human genome

We used the respective gencode annotation for each of the two versions of the human genomes: genocode 19 for hg19 and genocode 29 for hg38. We complemented the annotation of the genes with the information provided by PANTHER utilizing the Ensemble ID as the gene identifier. We removed all annotated elements (present in gencode) that were marked as unmapped IDs in PANTHER.

### Upset plot

All top candidate windows from the five populations (African, American, European, East Esian, South Asian) were pooled. Then for each window its presence/absence was computed for each population (Additional file 6: Table S7). Finally for each window the intersection was computed based on the presence/absence binary table. This table was then fed to the upset function of the UpSetR library [39] according to the reference manual and example.

### Rhesus macaque

We mapped the reads from 150 rhesus macaque individuals sampled from the Tulane National Primate Research Center, Covington, LA to the reference rhesus macaque genome Mmul_8 using BWA-mem with default parameters. These sequence data are described in **Petty et al. (2021; PMID 33386679)**. Subsequently, we identified candidate SVs using Manta [40] for each of the bam files separately. Next we computed the region of low mapping quality by extracting reads with MQ < 5 and generated a per sample region file by requiring 5 reads of MQ < 5 in order to define an interval. The per sample VCF was subsequently filtered by these intervals to account for mapping artifacts and repetitive regions. The resulting VCF files were analyzed and merged using SURVIVOR [41] merge requiring a SV to be at least 50 bp long and up to 1000 bp wobble on the start or stop breakpoint.

### Comparison of SVhound clSV candidates to hotspots in the 1000genomes data

We used the hotspots described by Lin and Gokcumen [26] and compared them to the candidate windows suggested by SVhound. We parsed the 1000genomes VCF file fixing the window size to 100 kb to have the same windows described in the hotspot analysis using vcf_autoparser_for_svhound_fix_windows.py. As the same genomic coordinates were used in both analyses, we compared their classification: “is it hotspot” from Lin and Gokcumen (number of SV ≥ 6) and “is it candidate” from SVhound (belongs to the top 1% windows with the highest probabilities of new SV) to then compute the intersection with a Venn diagram in R.

### Comparison of SVhound clSV candidates to rare alleles in the 1000genomes data

For each SV 1000genomes dataset we classified whether or not it contained a rare allele (MAF < 1%) using the AF tag from the VCF file using rareSV_detect_1kgp.py. Next, we associated each SV classified as having a rare allele with a genomic window dividing the genome into 100 kb windows using vcf_autoparser_for_svhound_fix_windows.py from the previous analysis. Finally, we used SVhound on the 100 kb windows and compared the windows containing rare SV with the clSV candidate windows to then compute the intersection with a Venn diagram in R.

## Supplementary Information

**Additional file 1:** **Fig. S1 - S10**.

**Additional file 2: Fig. S11.** Window length distribution.

**Additional file 3: Fig. S12.** Distribution of the number of detected SV-alleles for a fix sample size of 150, for the 1KGP data set.

**Additional file 4: Fig. S13.** Distribution of the number of detected SV-alleles for a fix sample size of 150, for the CCDG data set.

**Additional file 5: Fig. S14.** Distribution of the number of detected SV-alleles for a fix sample size of 150, for the Macaque data set.

**Additional file 6:** **Supplementary Tables 1 - 21**.

## Data Availability

Rhesus VCF files (https://github.com/lfpaulin/SVhound) and the R package contain the information of the sources used. 1000 genomes VCF file is available at: https://ftp.1000genomes.ebi.ac.uk/vol1/ftp/phase3/integrated_sv_map/ALL.wgs.mergedSV.v8.20130502.svs.genotypes.vcf.gz. CCDG data is available at: https://ftp.ncbi.nlm.nih.gov/pub/dbVar/data/Homo_sapiens/by_study/tsv/, nstd223.GRCh38.variant_call.tsv.gz, nstd223.GRCh38.variant_region.tsv.gz.

## Abbreviations

SVs: Structural Variations/Structural Variants
1KGP: 1000 Genomes Project
CCDG: Centers for Common Disease Genomics
SNV: Single Nucleotide Variations
CNV: Copy Number Variations
kbp: Kilobases
Mbp: Megabases
bp: Bases
GIAB: Genome in a Bottle
clSV: Clairvoyant SV
MAF: Minor Allele Frequency
QC: Quality Control
FP: False Positive
MHC: Major Histocompatibility Complex
AFS: Allele Frequency Spectrum
ESF: Ewens Sampling Formula
MQ: Mapping Quality
VCF: Variant Call Format
LINE: Long interspersed nuclear elements
LTR: Long terminal repeat
ERV: Endogenous retroviral sequence
DNA: Deoxyribonucleic acid
lncRNA: Long non-coding Ribonucleic acid
AFR: African
AMR: Admixed American
EUR: European
EAS: East Asian
SAS: South Asian
