# Supplementary material for: SVhound: detection of regions that harbor yet undetected structural variation
Source: BMC Bioinformatics. 2023 Jan 20;24:23. doi: 10.1186/s12859-022-05046-6 (PMC9854228; doi:10.1186/s12859-022-05046-6)
Supplement: Supplementary file 1 — Additional file 1: Fig. S1 - S10. [file 12859_2022_5046_MOESM1_ESM.docx]

**Supplement**

**Figures**

**
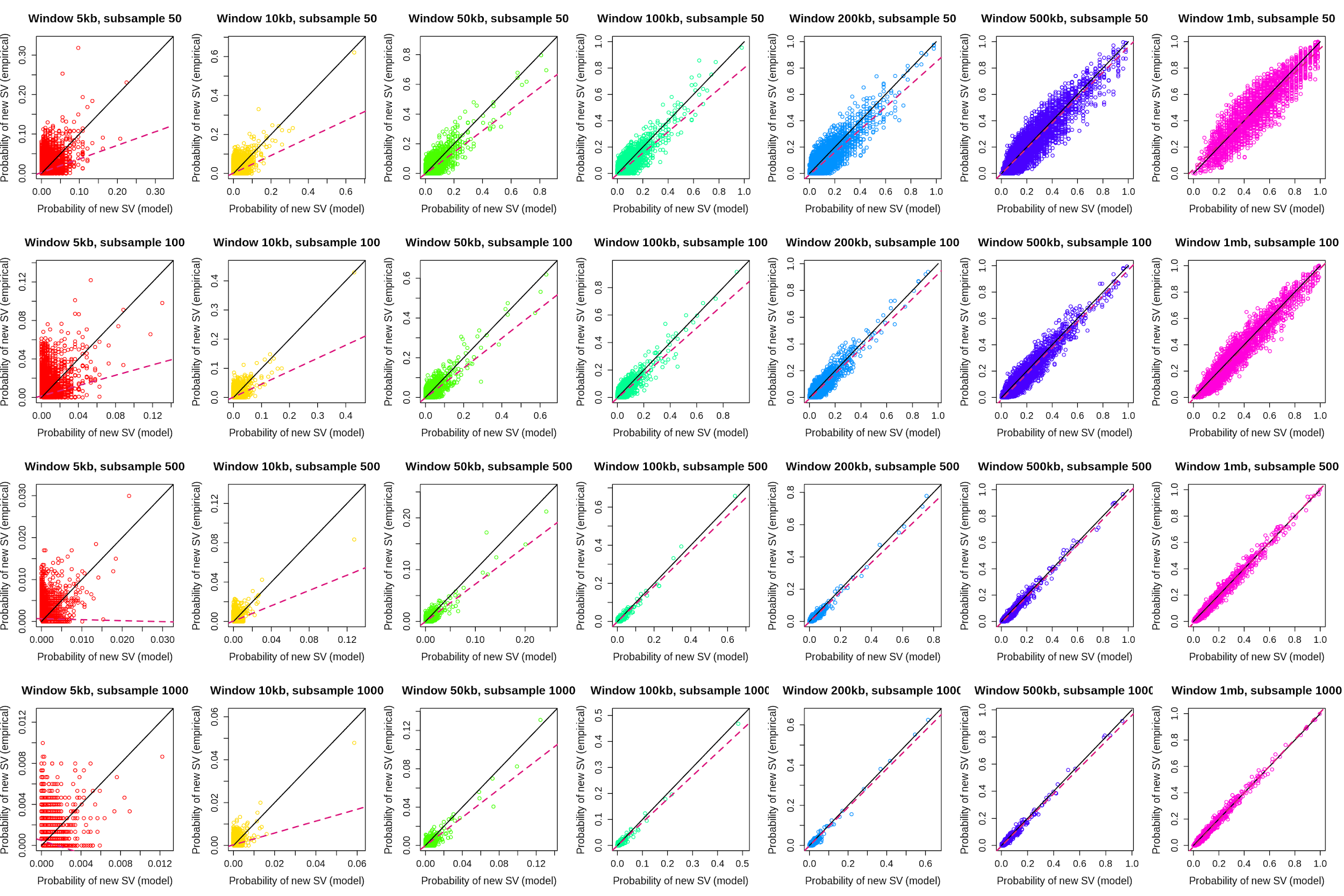
**

**Supplementary figure 1:**

*Performance of SVhound for distinct window and sample sizes. We compared the probability of detecting a new SV-allele* $p_{new}$ *to the fraction* $f_{undetected}$ *of SV-alleles that are present in the 1KGP data set, but were not sampled (see Methods). Here we show the scatterplots of a single benchmark instance (100 independent sampling procedures were performed for each combination of window and sample size). The dotted red line represents the regression line, and in solid black is the identity line.*

*
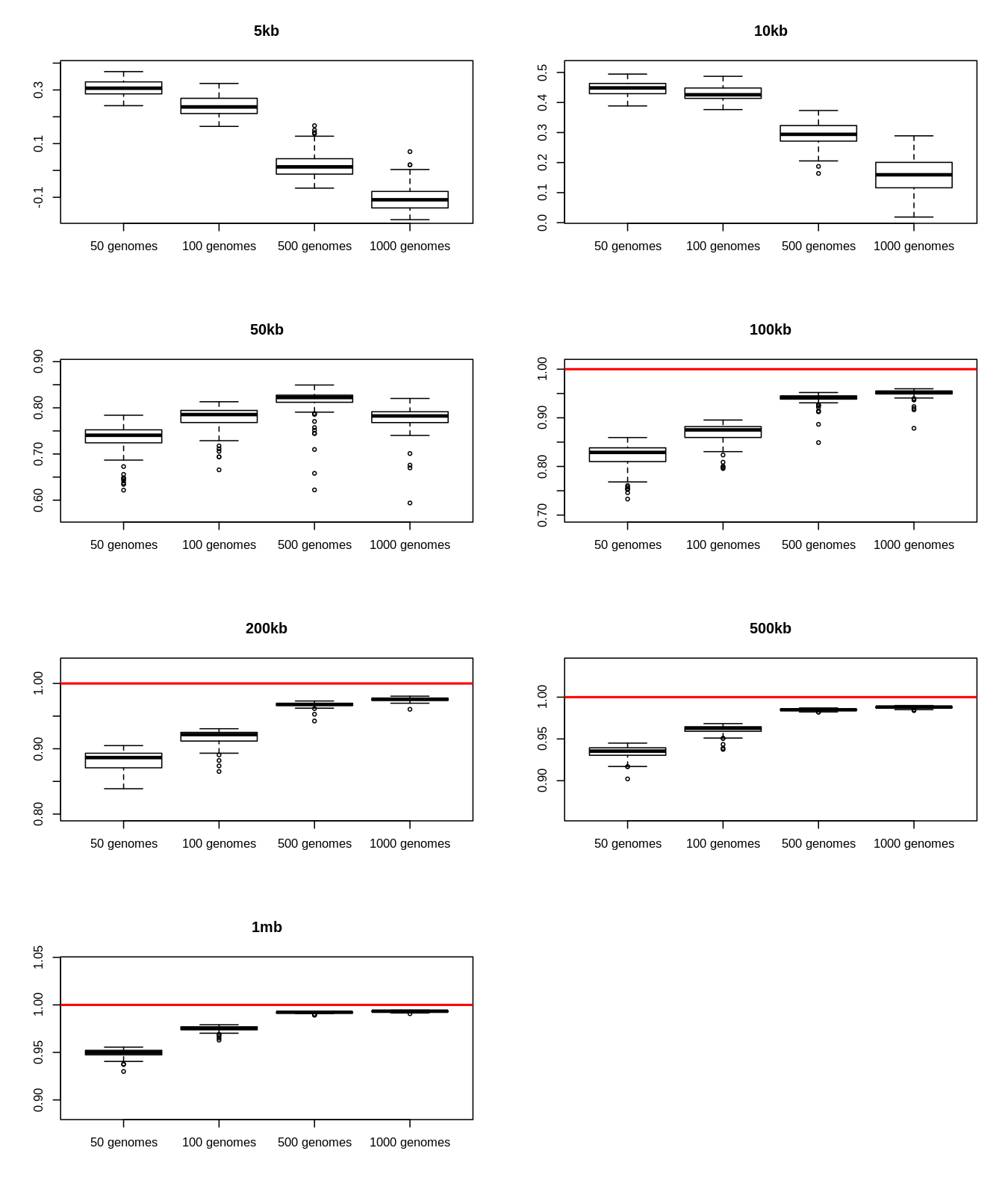
*

**Supplementary Figure 2:**

*Performance of SVhound for distinct window (each panel) and sample sizes (50, 100, 500 and 1,000 genomes). For the evaluation we compared the* $p_{new}$ *estimate with the fraction* $f_{undetected}$ *of SV-alleles that do not occur in the random sample but were observed in the full 1KGP data. Here we show the correlation coefficient (*$r$*, y-axis) of 100 independent replicates of the sampling procedures.*

*
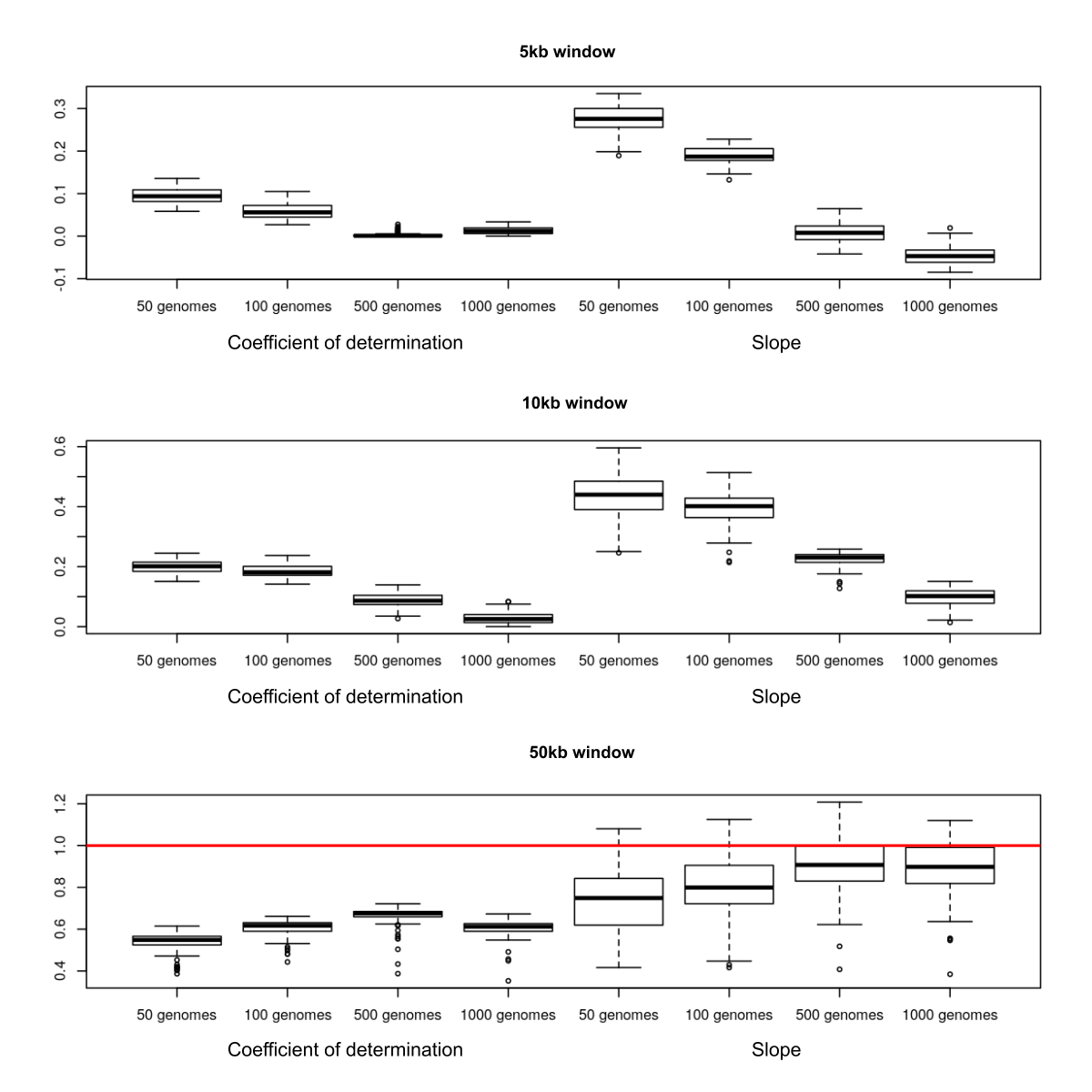
*

*
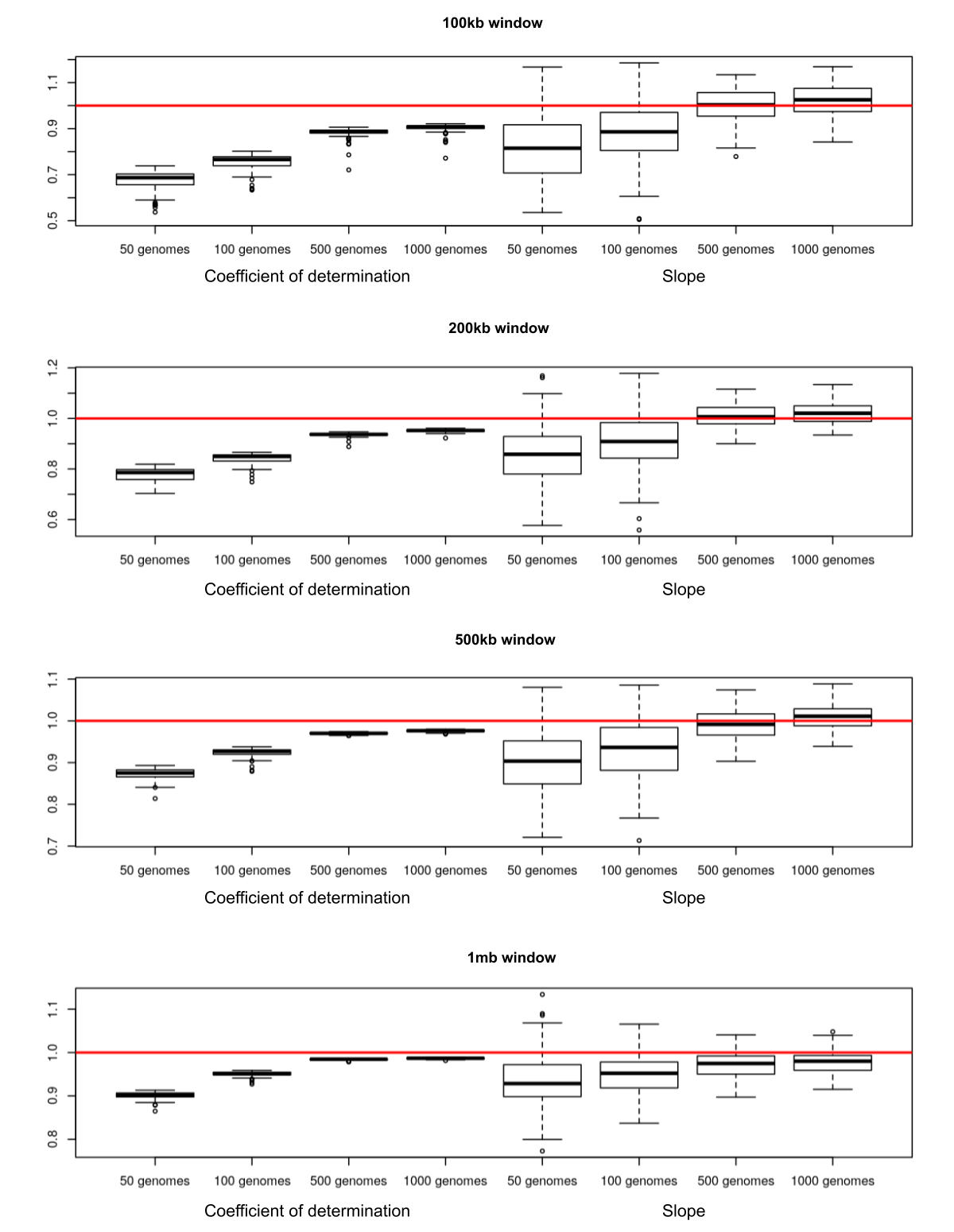
*

**Supplementary Figure 3:**

*Performance of SVhound for distinct window (panels) and sample sizes (50, 100, 500 and 1,000 genomes). For the evaluation we compared the* $p_{new}$ *estimate with the fraction* $f_{undetected}$ *of SV-alleles that do not occur in the random sample but were observed in the full 1KGP data. Here we show the coefficient of determination (*$r^{2}$*) and the slope of 100 independent replicates of the sampling procedures.*

*
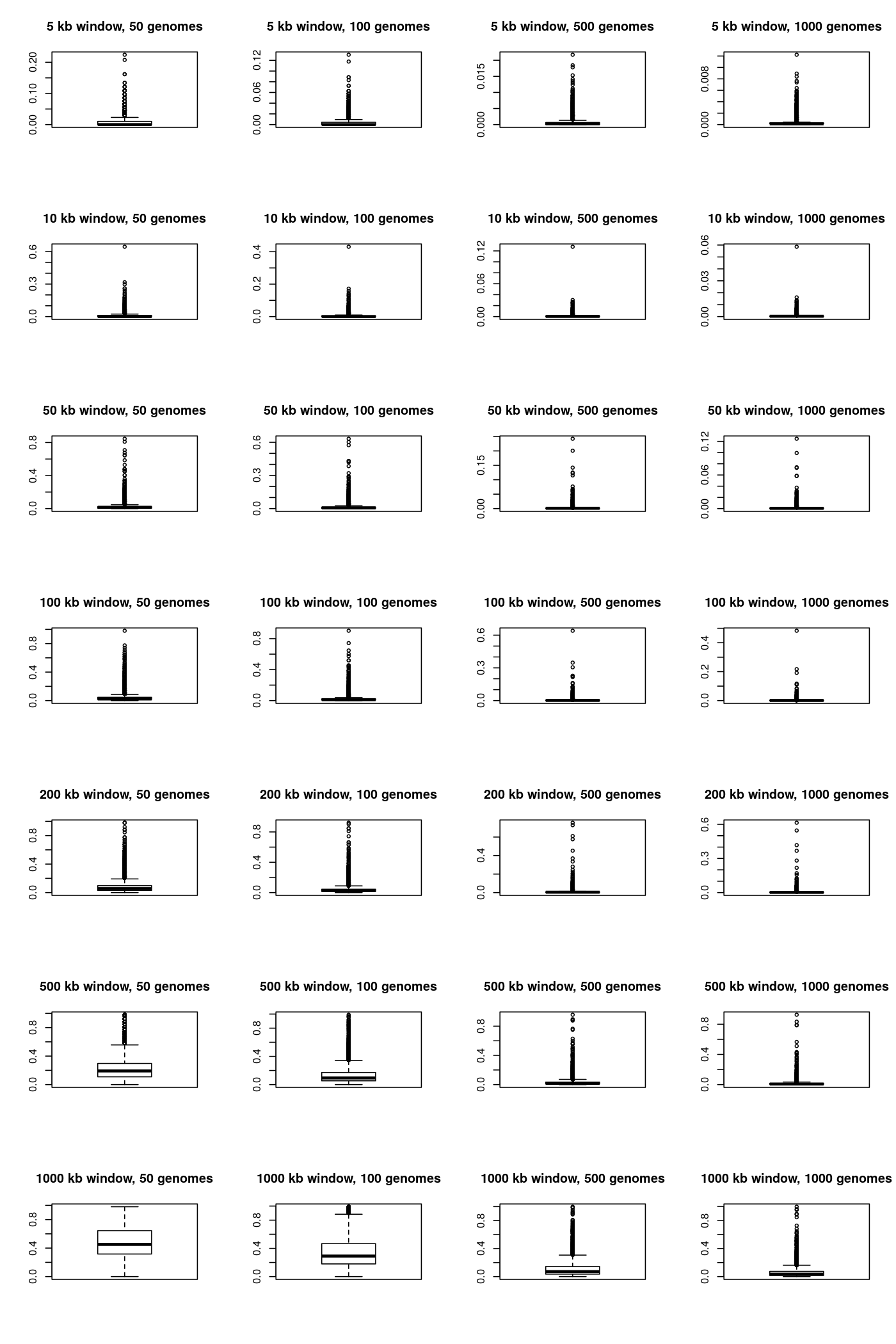
*

**Supplementary Figure 4:**

*Distribution of* $p_{new}$ *by window and sample size.*


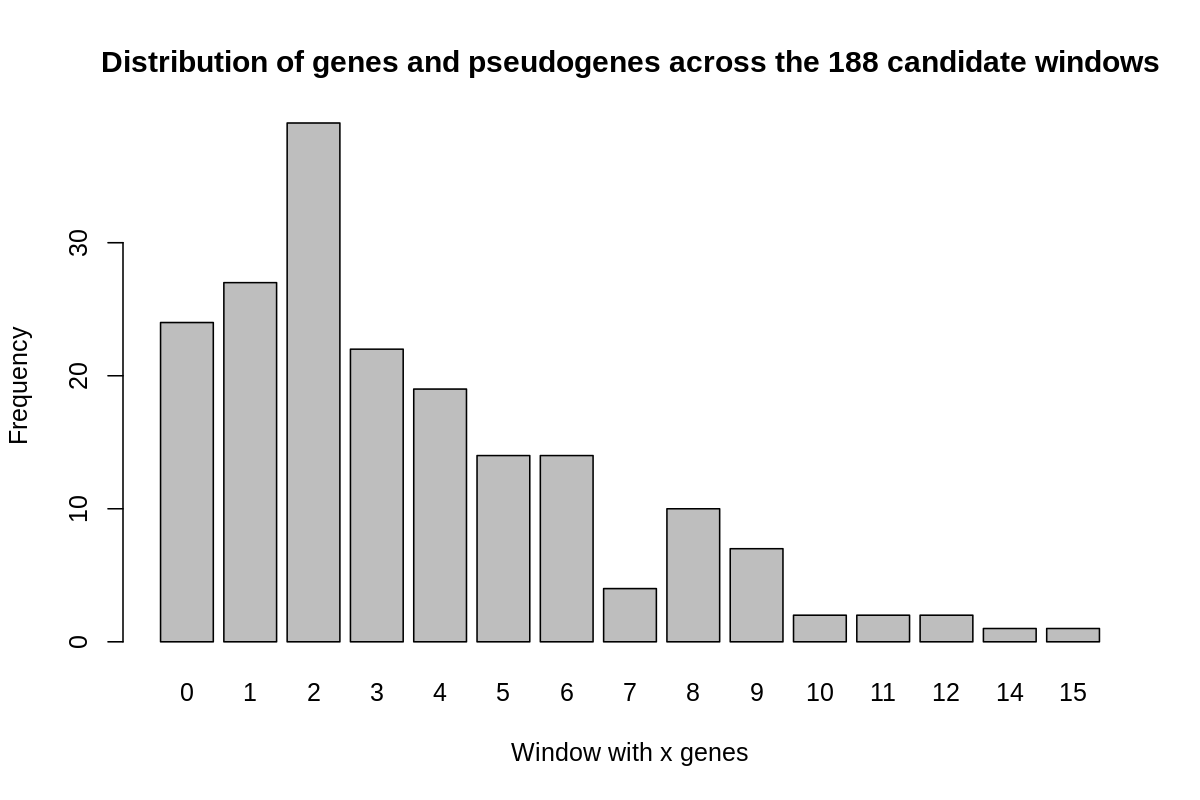


**Supplementary Figure 5:**

*Distribution protein coding genes, non-coding genes and pseudogenes in the 188 candidate windows of the 1KGP data set. Each bar represents the number of windows overlapping with 0,1,2,...15 genes or pseudogenes.*


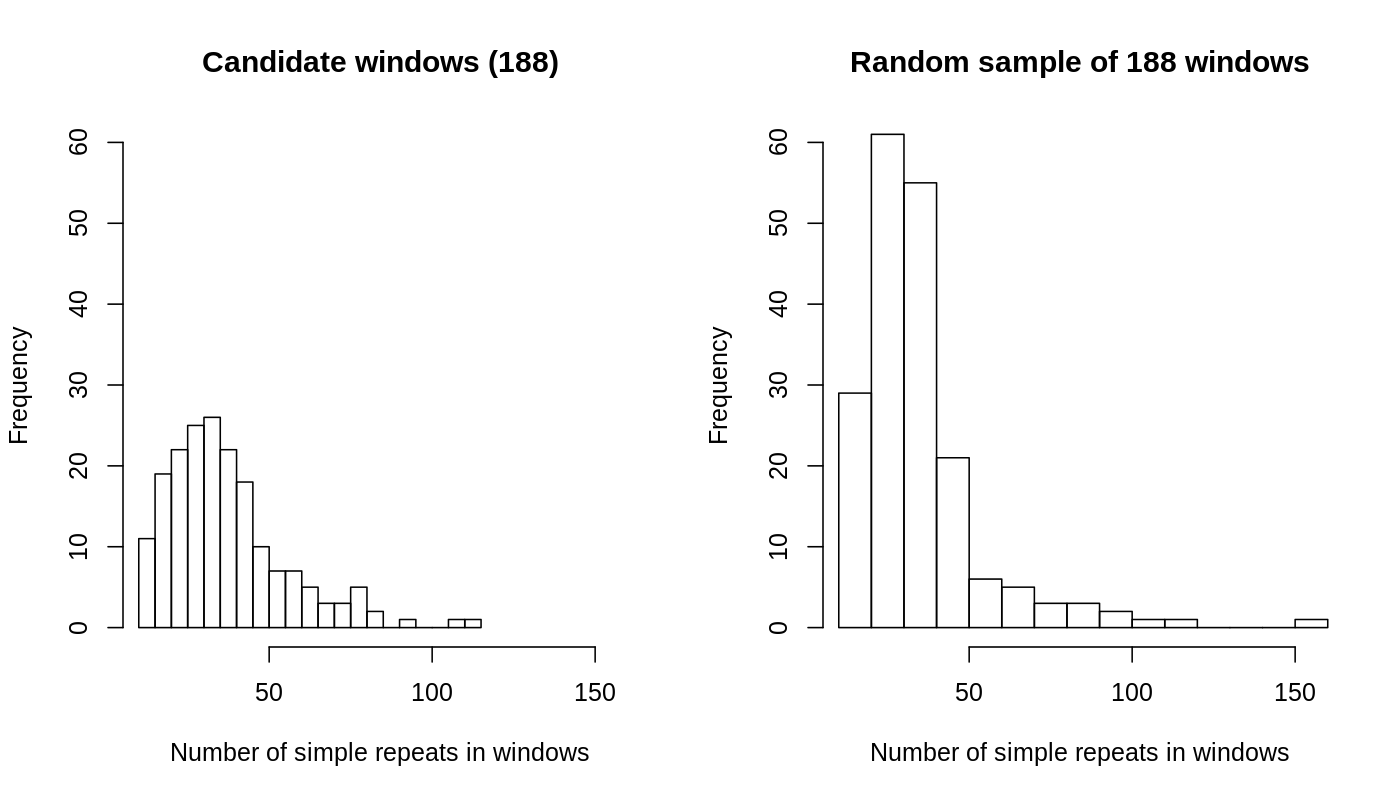


**Supplementary Figure 6:**

*Distribution simple tandem repeats across the 188 candidate windows (leff) and a random selection of 188 windows (right). T test = 1.0083, p-value=0.314.*

*KS test D=0.10638, p-value = 0.2378*

***
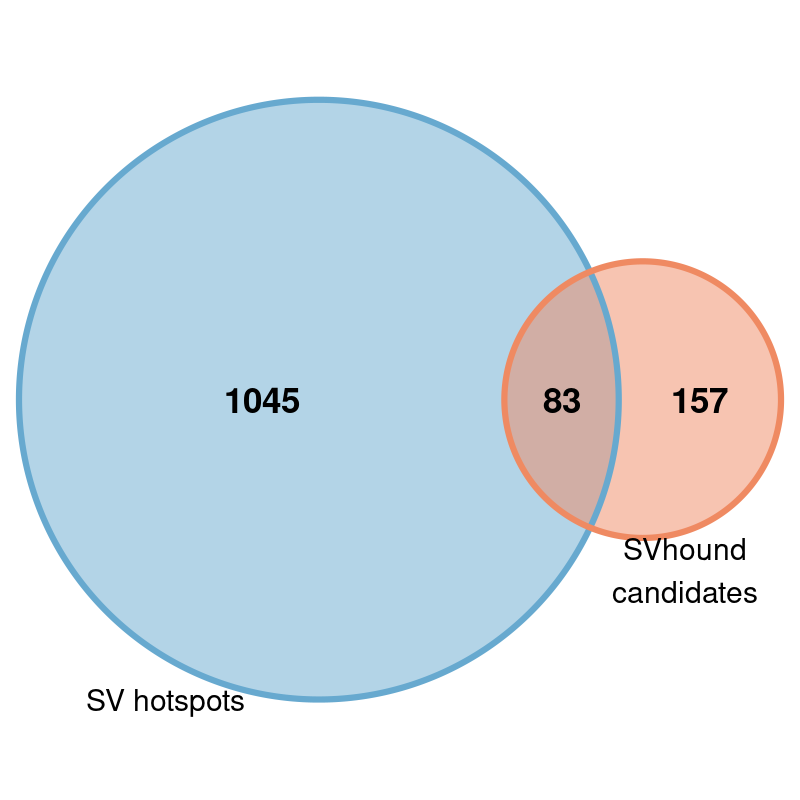
***

**Supplementary Figure 7:**

*Comparison between SVhound predicted regions. SVhound v. SV hotspots clearly highlight that SVhound includes some of the hotspots but also identifies 157 regions with high probability (min=0.25%, max=18%) to harbor additional SV, while the 377 windows solely marked as hotspots have a probability to harbor additional SV from 0.01% to 0.24%.*

*
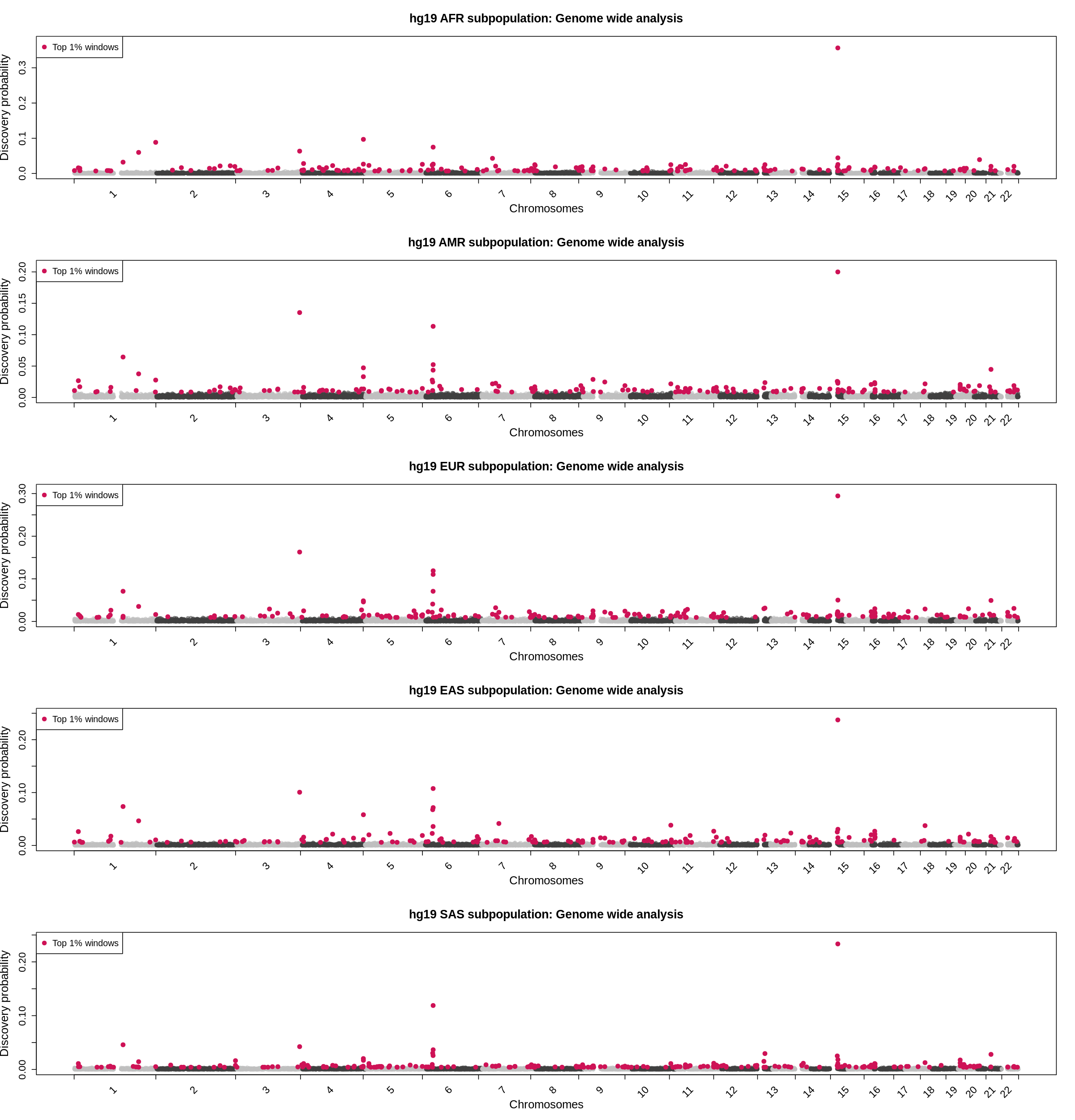
*

**Supplementary figure 8:**

*Genome wide analysis of the 1KGP data set when dividing the 2,504 genomes into their five ethnic groups according to the 1KGP super-population structure (African (AFR), Ad mixed American (AMR), European (EUR), East Asian (EAS), South Asian (SAS)). In red are shown the candidate windows along the 22 autosomes of the human genome, and in gray (alternating shades by chromosome) are shown the rest of the windows. The list of candidate windows by ethnic group is shown in* ***Supplementary Table 7****.*

*
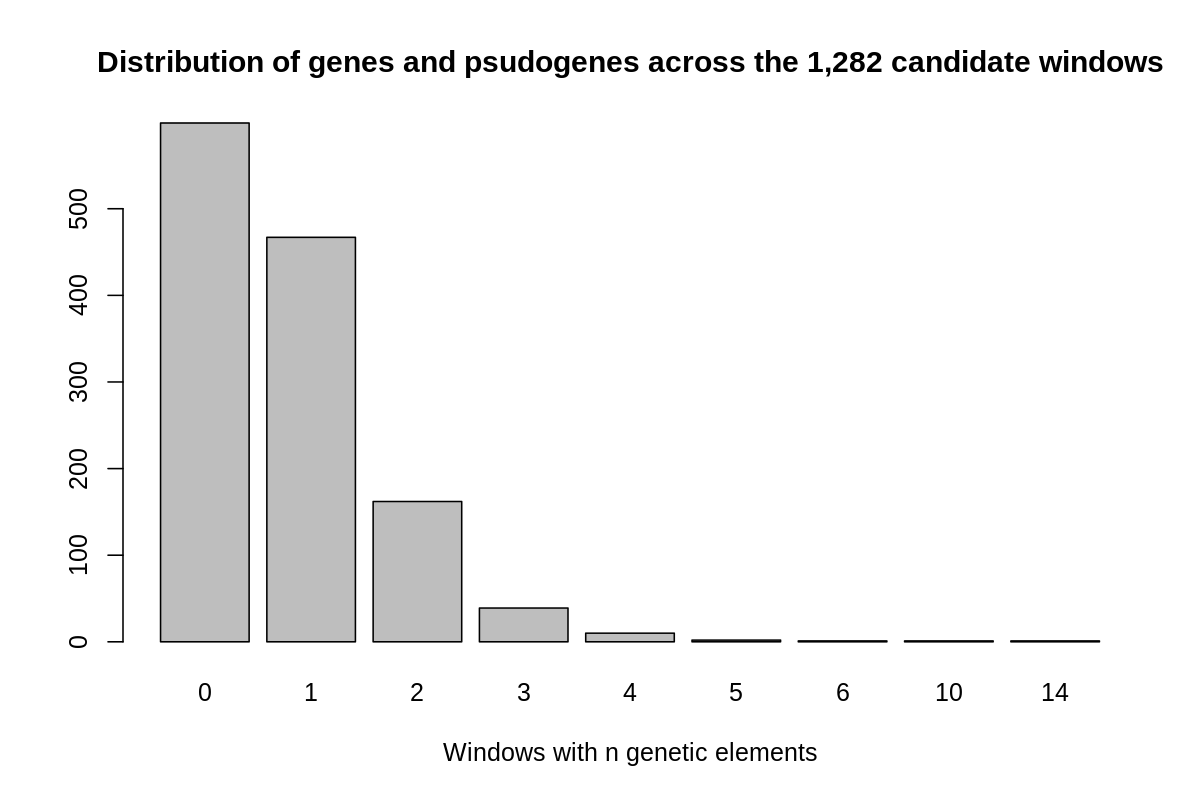
*

**Supplementary figure 9:**

*Distribution genes and pseudogenes in the 236 candidate windows of the CCDG data set. Each bar represents the number of windows overlapping with 0,1,2,...17 genes or pseudogenes.*

*
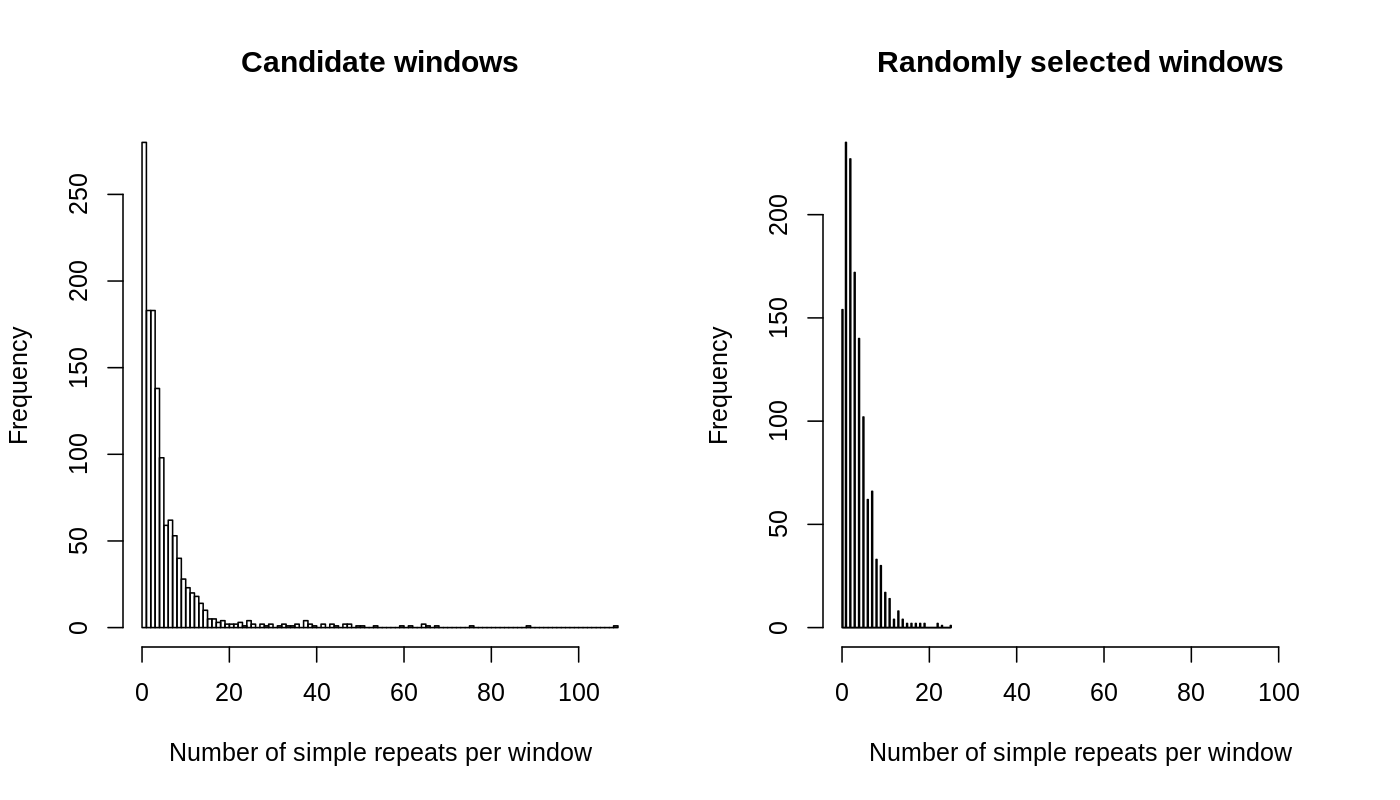
*

**Supplementary figure 10:**

*Distribution simple tandem repeats across the 1,282 candidate windows (leff) and a random selection of 1,282 windows (right). T test = 9.2169, p-value < 2.2e-16*

*KS test D = 0.1209, p-value = 1.453e-08*

File: Supplemet-figure-11.pdf

**Supplementary figure 11:**

*Distribution of the window size length when only including 10-50% of the SV and 150 individuals for the CCDG dataset.*

File: Supplemet-figure-12.pdf

**Supplementary figure 12:**

*Distribution of the number of different SV-alleles, k, for 100 subsamples of 150 individuals of the 1KGP dataset. Notice that the estimated window size may be different for each replicate*

File: Supplemet-figure-13.pdf

**Supplementary figure 13:**

*Distribution of the number of different SV-alleles, k, for 100 subsamples of 150 individuals of the CCDG dataset. Notice that the estimated window size may be different for each replicate*

File: Supplemet-figure-14.pdf

**Supplementary figure 14:**

*Distribution of the number of different SV-alleles, k, the 150 individuals of the rhesus dataset*
