# Supplementary figures and images for: SVhound: detection of regions that harbor yet undetected structural variation
Source: BMC Bioinformatics. 2023 Jan 20;24:23. doi: 10.1186/s12859-022-05046-6 (PMC9854228; doi:10.1186/s12859-022-05046-6)

CCDG 150 indiv, 10%

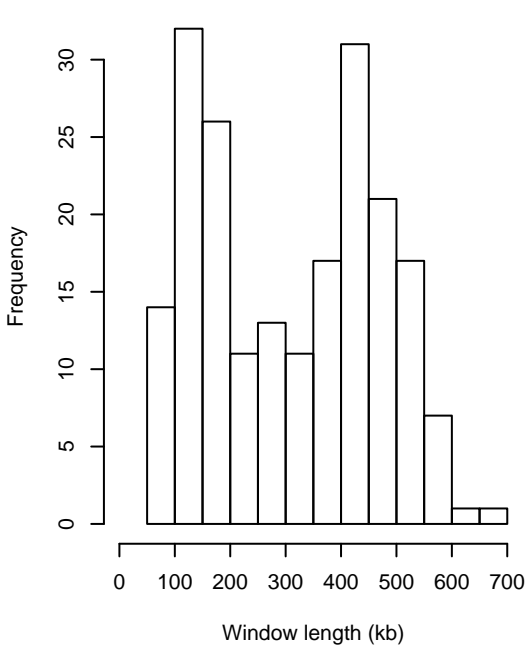

CCDG 150 indiv, 20%

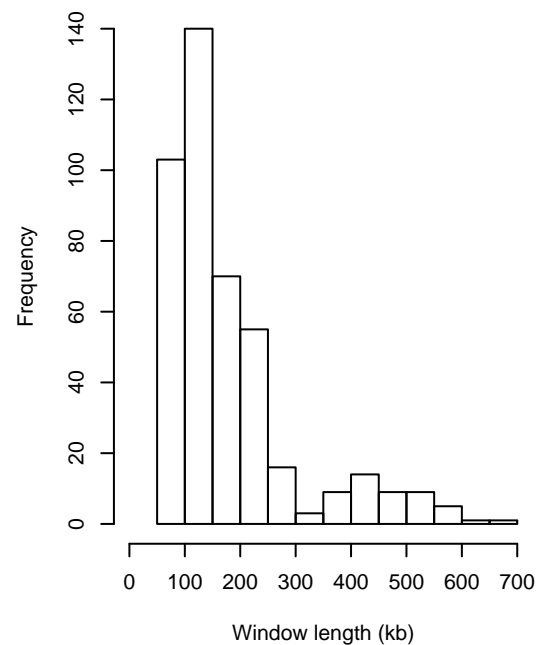

CCDG 150 indiv, 30%

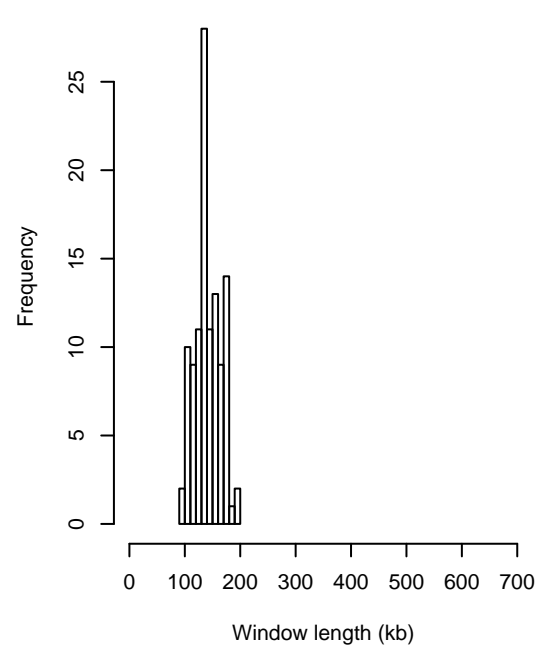

CCDG 150 indiv, 40%

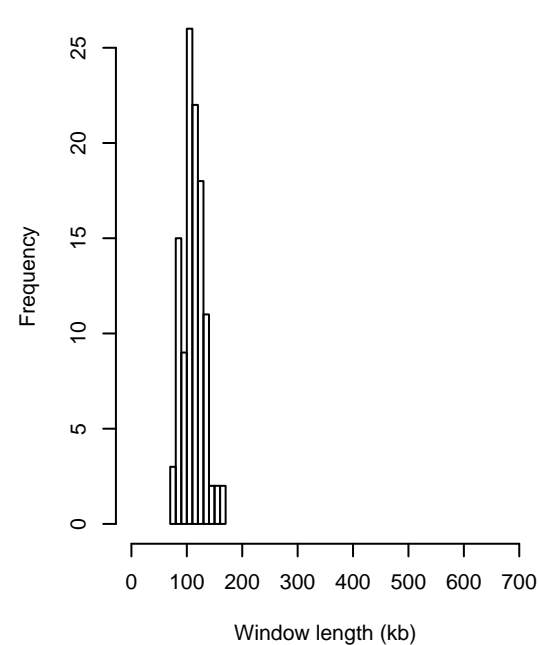

CCDG 150 indiv, 50%

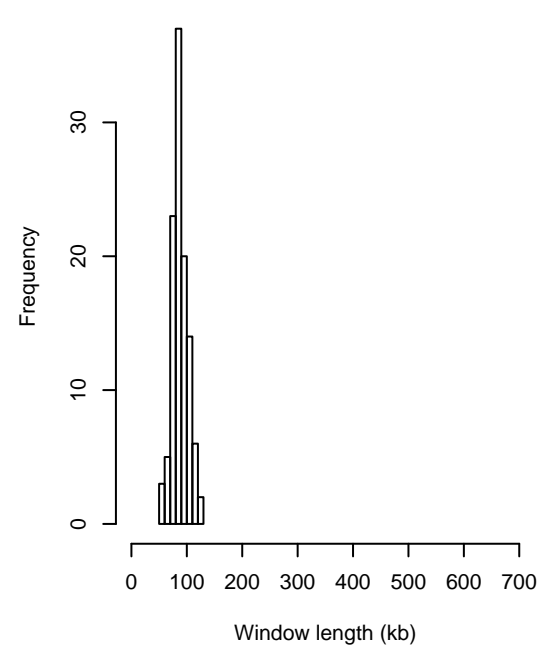

Supplement: Supplementary file 2 — Additional file 2: Fig. S11. Window length distribution. [file 12859_2022_5046_MOESM2_ESM.pdf]

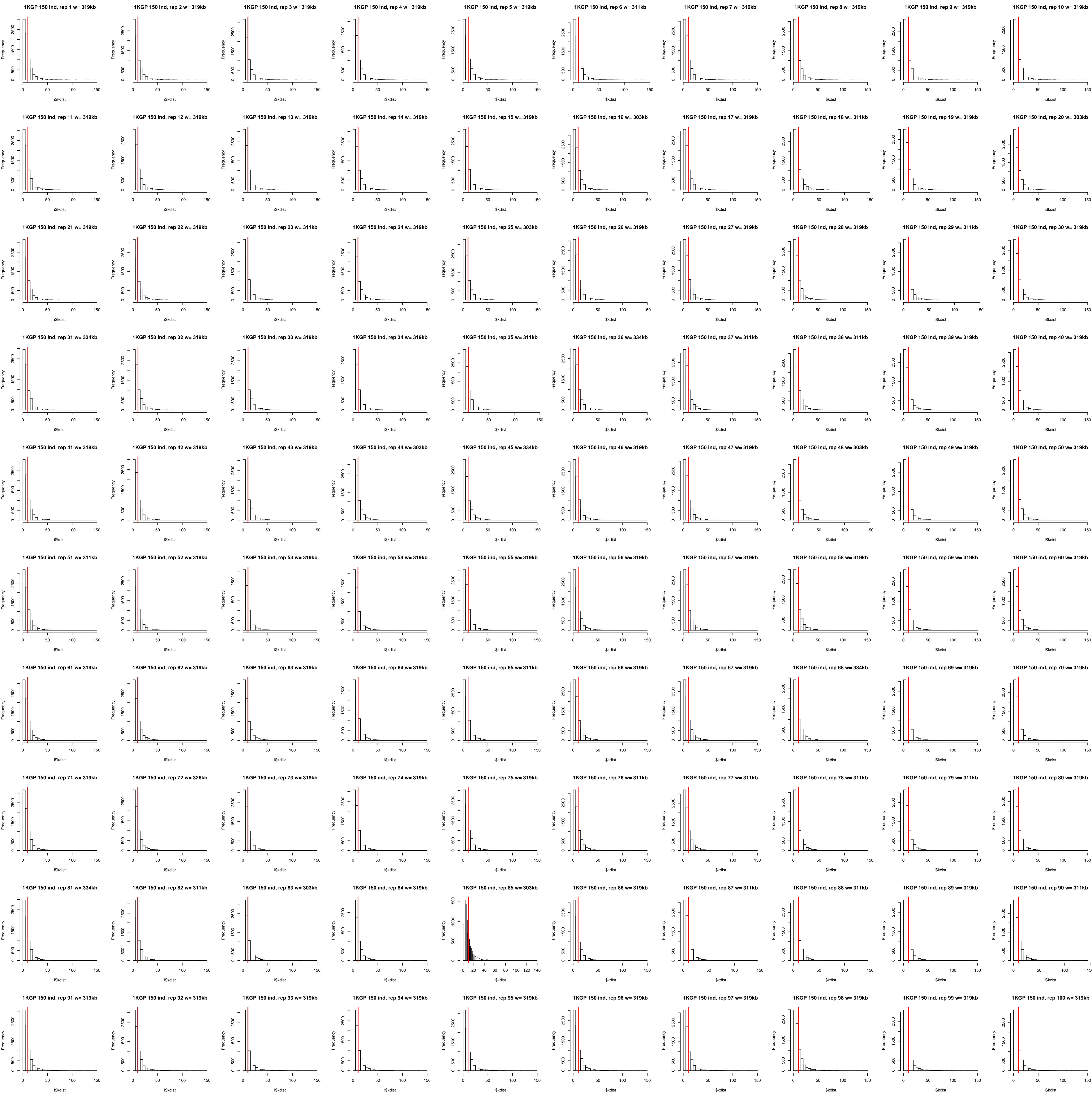

Supplement: Supplementary file 3 — Additional file 3: Fig. S12. Distribution of the number of detected SV-alleles for a fix sample size of 150, for the 1KGP data set. [file 12859_2022_5046_MOESM3_ESM.pdf]

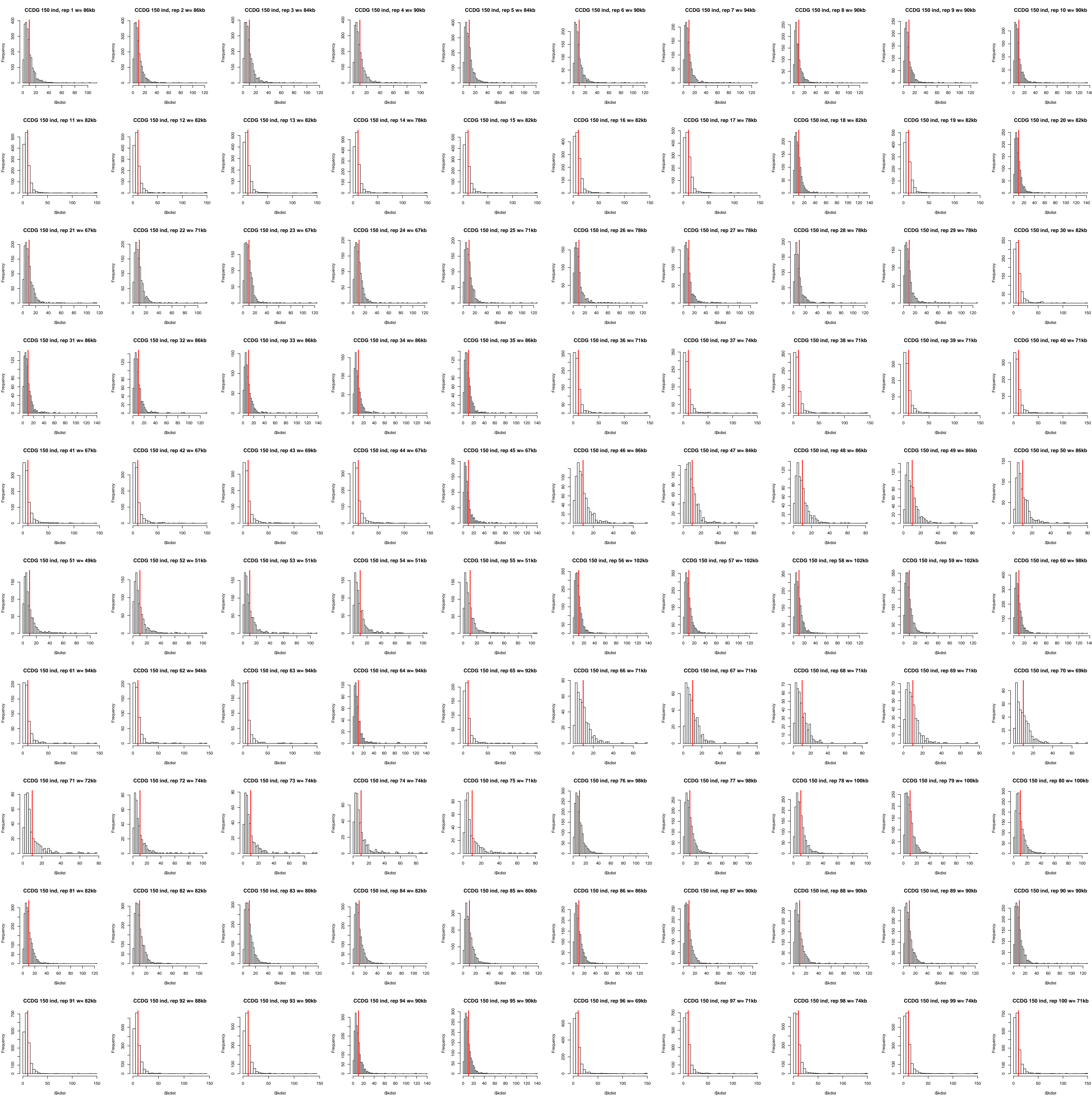

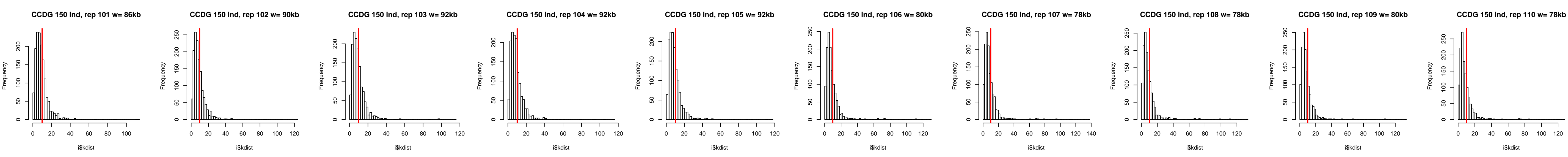

Supplement: Supplementary file 4 — Additional file 4: Fig. S13. Distribution of the number of detected SV-alleles for a fix sample size of 150, for the CCDG data set. [file 12859_2022_5046_MOESM4_ESM.pdf]

## Rhesus 150 ind, w= 27kb

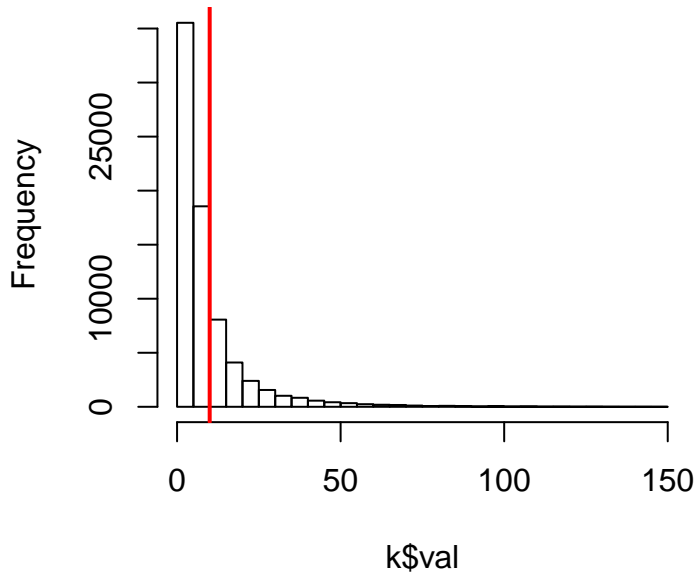

Supplement: Supplementary file 5 — Additional file 5: Fig. S14. Distribution of the number of detected SV-alleles for a fix sample size of 150, for the Macaque data set. [file 12859_2022_5046_MOESM5_ESM.pdf]
